# A large-scale transcontinental river system crossed West Antarctica during the Eocene

**DOI:** 10.1126/sciadv.adn6056

**Published:** 2024-06-05

**Authors:** Maximilian Zundel, Cornelia Spiegel, Chris Mark, Ian Millar, David Chew, Johann Klages, Karsten Gohl, Claus-Dieter Hillenbrand, Yani Najman, Ulrich Salzmann, Werner Ehrmann, Jürgen Titschack, Thorsten Bauersachs, Gabriele Uenzelmann-Neben, Torsten Bickert, Juliane Müller, Rober Larter, Frank Lisker, Steve Bohaty, Gerhard Kuhn, the Science Team of Expedition PS104

**Affiliations:** ^1^ Faculty of Geosciences, University of Bremen, Bremen, Germany.; ^2^ School of Earth Sciences, University College Dublin, Belfield, Dublin, Ireland.; ^3^ British Geological Survey, Keyworth, Nottingham, UK.; ^4^ Department of Geology, Trinity College Dublin, College Green, Dublin, Ireland.; ^5^ Department of Geosciences, Alfred Wegener Institute, Helmholtz Center for Polar and Marine Research, Bremerhaven, Germany.; ^6^ British Antarctic Survey, Cambridge, UK.; ^7^ Lancaster University, Lancaster Environment Centre, Lancaster, UK.; ^8^ Department of Geography and Environmental Sciences, Northumbria University, Newcastle upon Tyne, UK.; ^9^ Institute for Geophysics and Geology, University of Leipzig, Leipzig, Germany.; ^10^ MARUM–Center for Marine Environmental Sciences, Bremen, Germany.; ^11^ Marine Research Department, Senckenberg am Meer, Wilhelmshaven, Germany.; ^12^ Institute of Organic Biogeochemistry in Geo-Systems, RWTH Aachen University, Aachen, Germany.; ^13^ Institute of Earth Sciences, University of Heidelberg, Heidelberg, Germany.

## Abstract

Extensive ice coverage largely prevents investigations of Antarctica’s unglaciated past. Knowledge about environmental and tectonic development before large-scale glaciation, however, is important for understanding the transition into the modern icehouse world. We report geochronological and sedimentological data from a drill core from the Amundsen Sea shelf, providing insights into tectonic and topographic conditions during the Eocene (~44 to 34 million years ago), shortly before major ice sheet buildup. Our findings reveal the Eocene as a transition period from >40 million years of relative tectonic quiescence toward reactivation of the West Antarctic Rift System, coinciding with incipient volcanism, rise of the Transantarctic Mountains, and renewed sedimentation under temperate climate conditions. The recovered sediments were deposited in a coastal-estuarine swamp environment at the outlet of a >1500-km-long transcontinental river system, draining from the rising Transantarctic Mountains into the Amundsen Sea. Much of West Antarctica hence lied above sea level, but low topographic relief combined with low elevation inhibited widespread ice sheet formation.

## Teaser

Before glaciation, West Antarctica was a vast lowland crossed by rivers routed in the >1500-km distant Transantarctic Mountains.

## INTRODUCTION

**Fig. 1.**
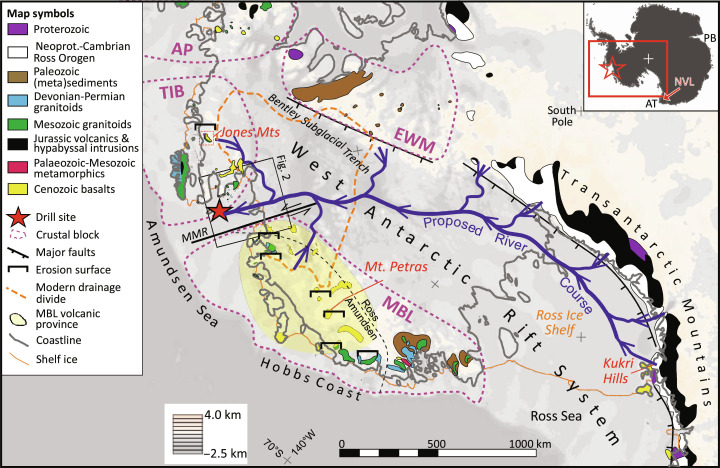
Overview map of West Antarctica. The map shows the subglacial topography of the study area (*68*), including major geological units and tectonic structures (*5*, *8*, *13*, *73*) as well as the proposed (schematic) location of the Eocene river system. Inset map shows the position of the figure on the Antarctic continent. The black square refers to the location of the map in Fig. 2. Abbreviations: AP, Antarctic Peninsula; AT, Adare Trough; EWM, Ellsworth-Whitmore Mountains; MBL, Marie Byrd Land; MMR, Mt. Murphy Rift; NVL, North Victoria Land; PB, Prydz Bay; TIB, Thurston Island Block.

**Fig. 2.**
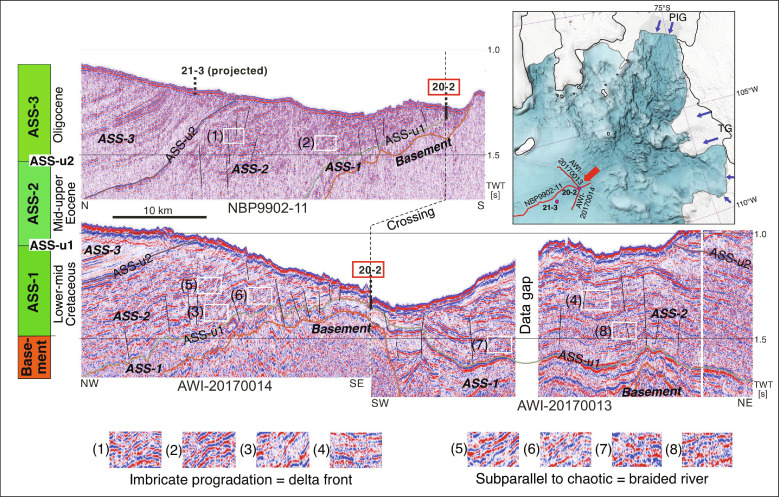
Intersecting seismic profiles from the middle shelf of the eastern Amundsen Sea Embayment with seismic characteristics indicating a fluvial braided river or deltaic system in middle to late Eocene seismic unit ASS-2. The left side shows a schematic stratigraphic column with the stratigraphic units and unconformities interpreted from the seismic profiles. Site PS104_20-2 (abbreviated as “20-2”), from which the Polarstern Sandstone was drilled, is indicated by red boxes and a red arrow. The regional unconformity ASS-u1 (*21*) is also indicated. Numbered examples in white boxes are described in the legend. The seismic horizons and units are adopted from Gohl *et al.* (*21*) with ages modified to lower to middle Cretaceous for unit ASS-1, middle to upper Eocene for unit ASS-2, and Oligocene for unit ASS-3 according to the stratigraphy of the MeBo70 drill cores (*4*, *20*; this study). Thin black lines mark interpreted faults. MeBo70 drill site PS104_20-2 (containing the Polarstern Sandstone) is located at the intersection of the three seismic profiles, while the position of site PS104_21-3 [containing Early Oligocene strata (*20*)] is projected from the real drill location ~5 km to the west. IBCSO-v2 bathymetric map [upper right; (*74*)] shows the locations of the seismic profiles and the MeBo70 drill sites as well as the modern flow directions of Pine Island Glacier, Thwaites Glacier, and neighboring glaciers (blue arrows). The coastlines are delineated by black lines, and the ice shelves are delineated by gray lines. Abbreviations: PIG, Pine Island Glacier; TG, Thwaites Glacier.

**Fig. 3.**
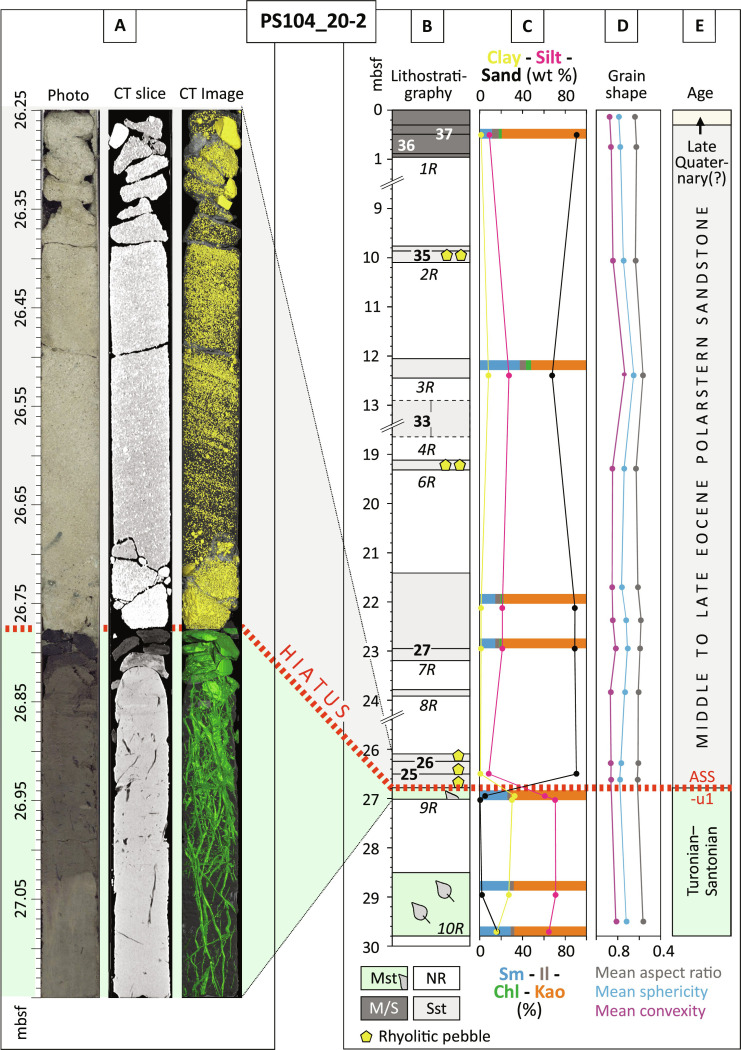
Lithostratigraphic record, sediment characteristics and clay mineralogy from the PS104_20-2 drill site. (**A**) Photo, computed tomography (CT) slice, and CT image of core 9R, covering the hiatus between the dark colored late Cretaceous mudstone containing a network of rootlets and the light colored Polarstern Sandstone. (**B**) Lithology of drilled intervals with recovery (gray and green shaded areas with italic labels marking core IDs) and positions of detrital geochronology samples (bold numbers) and rhyolite clast samples (yellow polygons). (**C**) Clay mineral composition of the fraction of <2 μm and sandstone grain size (sand versus silt versus clay). (**D**) Various grain-shape parameters of the <2-mm fraction [using the dynamic image analysis QICPIC technique; (*75*)]. (**E**) Stratigraphy. The regional unconformity ASS-u1 (*21*) is also indicated. The gray colored column above the unconformity represents the Polarstern Sandstone. Abbreviations: Chl, chlorite; Il, illite; Kao, kaolinite; mbsf, meters below sea floor; M/S, mud/sand; Mst, mudstone; NR, no recovery; Sst, sandstone; Sm, smectite.

Major Antarctic glaciation initiated at the Eocene-Oligocene transition ~34 million years ago (Ma), marking one of the most pronounced climate transitions of Phanerozoic times (*1*). The reconstruction of Antarctica’s paleoenvironmental and paleotectonic conditions before this transition, i.e., in the middle to late Eocene, provides important boundary conditions for understanding the subsequent cooling and onset of glaciation with major consequences for ice sheet modeling (*2*). However, geological records of middle to late Eocene environmental conditions in West Antarctica are particularly sparse because no sedimentary rocks of that age crop out in the area, which, today, is almost entirely ice-covered. At most locations on the continental shelf, overconsolidated subglacial till deposited during the Last Glacial Maximum (~20,000 years ago) and earlier glacial periods and/or indurated old sedimentary strata have hampered the recovery of pre-Quaternary sediments by conventional coring techniques. Deployment of the remotely operated Seafloor Drill Rig MARUM-MeBo70 (*3*) during *RV Polarstern* Expedition PS104 in 2017 retrieved Cretaceous to Neogene sedimentary strata from the inner and middle shelf of the Amundsen Sea Embayment (Fig. 1) (*3*, *4*). We report results from drill site PS104_20-2, which targeted the southernmost and oldest sedimentary sequences on the Amundsen Sea Embayment shelf (Fig. 2). The lower part of the drill core comprises Cretaceous (93 to 83 Ma) mudstone bearing fossil roots and diverse pollen and spores that indicate the existence of a late Cretaceous temperate rainforest (*4*). The Cretaceous strata are overlain by a thin layer of indurated lignite fragments, which, in turn, is overlain by 17 to 24 m of gravelly sandstone of Eocene age (Fig. 3, A, B, and E) (*4*). For this study, we investigated the Eocene sandstone, which we name “Polarstern Sandstone.” The Polarstern Sandstone is barren of any (micro)fossils that may provide information on the Eocene paleoenvironment. We applied geochronological, thermochronological, and isotope analyses to heavy minerals contained in the sandstone, combined with the analysis of the clay mineral composition of its matrix, for obtaining information on the provenance of the Polarstern Sandstone. The complementary use of petrography and lipid biomarkers provided additional information to constrain the conditions associated with sediment deposition. Combined, our data allow for reconstructing West Antarctica’s Eocene landscape prior to large-scale permanent glaciation.

The Amundsen Sea Embayment is situated between two continental fragments of West Antarctica, namely, Marie Byrd Land and the Thurston Island Block (Fig. 1). Both are dominated by Mesozoic magmatic rocks, which were truncated by the low-relief West Antarctic Erosion Surface (WAES) representing a major erosive period during or after late Cretaceous times (*5*, *6*). The WAES was later dissected by differential uplift and can hence be traced today at different elevations over large parts of West Antarctica (*7*). Marie Byrd Land and the Thurston Island Block are separated by a branch of the West Antarctic Rift System, the Mt. Murphy Rift (*8*). The West Antarctic Rift System resulted from the separation of East from West Antarctica, which led to seafloor spreading in the Adare Trough in the outer Ross Sea between ~43 and 26 Ma (*9*). The development of the West Antarctic Rift System was accompanied by (still ongoing) alkaline volcanic activity of the Marie Byrd Land volcanic province (Fig. 1), whose main period has been dated at ~30 to 28 Ma (*10*, *11*). The oldest volcanic rocks occur in the center of Marie Byrd Land, becoming younger toward the coast (*12*).

The inland shoulder of the West Antarctic Rift System is formed by the Transantarctic Mountains, which today reach elevations of >4000 m above sea level, representing the uplifted edge of East Antarctica. Basement rocks exposed in the Transantarctic Mountains were affected by several periods of Proterozoic mountain building (*13*). However, most exposed rocks result from magmatism, metamorphism, and erosion of the Late Proterozoic to Cambrian Ross Orogen (~590 to 480 Ma) (*13*). These exposures were later intruded and capped by the magmatic rocks of the Jurassic Ferrar Large Igneous Province, which formed at ~180 Ma in response to the breakup of the Gondwana supercontinent (*13*). The Transantarctic Mountains started to rise during the late Eocene to early Oligocene, concomitant with erosion of up to 3.4 km within a relatively short interval of ~5 million years (Myr) (*14*).

Today, the West Antarctic Rift System hosts the West Antarctic Ice Sheet (WAIS). The main glacial systems of the WAIS draining into the Amundsen Sea are the Thwaites and the Pine Island Glaciers. The drill core, which contains the Polarstern Sandstone, was obtained from site PS104_20-2, situated within the glacial trough in front of the Pine Island Glacier, close to its glacial front (Figs. 1 and 2).

## RESULTS

### Sediment petrography and clay mineralogy

The Polarstern Sandstone at site PS104_20-2 shows rarely cross-stratification, upward-fining sequences, as well as high aspect ratio, sphericity, and convexity values of the sand grains (Fig. 3D, fig. S1, and Supplementary Tables). The sand grains consist predominantly of quartz (80 to 90%) but also include other minerals, such as zircon, apatite, and rutile, although heavy mineral contents and, particularly, apatite yields are generally low (<0.01%). About 30% of the apatite grains show naturally formed etching features on external and internal crystal surfaces, revealing fission tracks and other crystallographic defects (fig. S2). The most common type of pebbles (2 to 63 mm in diameter) contained in the sandstone is a white rhyolite (SiO_2_-rich volcanic rock), which is ≤5 cm in size (fig. S3). The white color is due to the alteration of the mineral feldspar to the clay mineral kaolinite. In addition, the sandstone contains granitic and quartzitic pebbles and a conspicuous pebble type, which we identified as a lithic arkose, formed by angular clasts embedded in a fine-grained, iron-rich matrix (fig. S4). The clay fraction (<2 μm) in the matrix of the sandstone is dominated by the mineral kaolinite (52 to 79%), followed by smectite (11 to 37%), illite (1 to 7%), and chlorite (0–5%) (Fig. 3C and Supplementary Tables).

### Radiogenic dating and (isotope) geochemistry

We analyzed (i) detrital zircon grains contained in the Polarstern Sandstone and zircon crystals included in the rhyolitic pebbles for Hf isotopes and U-Pb dates, (ii) detrital apatite grains for trace element systematics, Nd isotopes and U-Pb, and fission track dates, and (iii) detrital rutile grains for U-Pb dates. For statistically distinguishing different age groups from the detrital data, we used kernel density estimations. In addition to the offshore drill core samples, we obtained U-Pb dates from three onshore bedrock samples from the Jones Mountains. The Jones Mountains, situated on the Thurston Island Block (Fig. 1), are one of the few sites in West Antarctica where Cretaceous rhyolitic rocks are exposed (*15*) and, thus, may have been the source for the rhyolitic pebbles found in the Polarstern Sandstone.

**Fig. 4.**
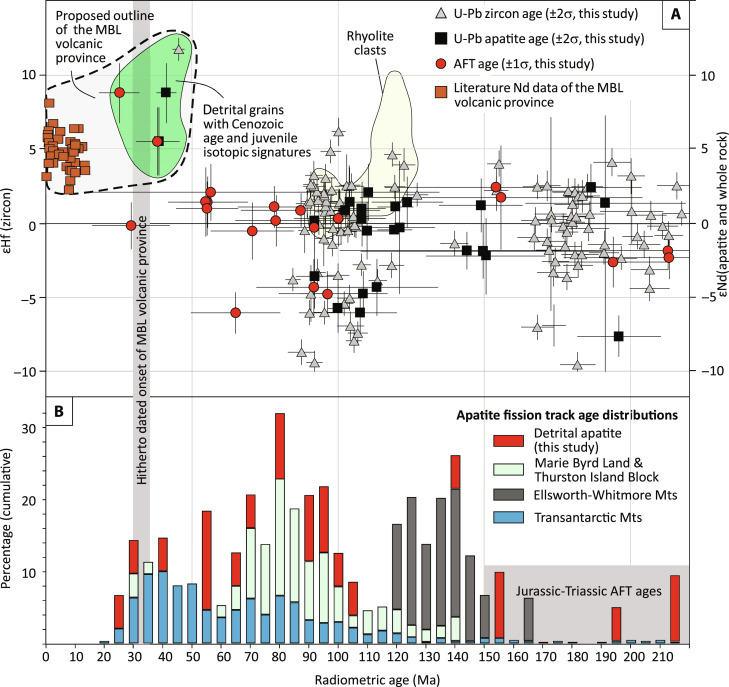
Isotopic and radiometric age data from detrital zircon and apatite contained in the Polarstern Sandstone. (**A**) Zircon Hf and apatite Nd isotopic data, plotted against their corresponding U-Pb or AFT ages. Detrital zircon and apatite grains with Cenozoic U-Pb and fission track ages (green shaded field) show juvenile Hf and Nd isotopic signatures. These are similar to published data from the Marie Byrd Land (MBL) volcanic province (*76*–*78*), which are shown for comparison. The yellow field shows the range of zircon U-Pb dates and corresponding Hf values from rhyolitic pebbles contained in the Polarstern Sandstones (figs. S6 and S8 and table S4), overlapping with data from the detrital zircons of the sandstone. Uncertainty bars for the isotopic data refer to 2SE (2*standard error, representing internal precision in ε units). (**B**) AFT data from MBL, the Thurston Island block, Ellsworth-Whitmore Mountains, and the Transantarctic Mountains compiled from the literature (*6*, *8*, *14*, *22*, *23*, *49*–*62*, *79*, *80*), compared to fission track dates of detrital apatite contained in the Polarstern Sandstone. The gray shaded field highlights the range of AFT ages, which are common in the Transantarctic Mountains and the Ellsworth-Whitmore Mountains but absent in MBL and the Thurston Island Block. Note that the AFT data from the literature are whole sample ages (usually the average of ages obtained from ~20 single grains), whereas the detrital grain dates obtained for this study (shown in red) refer to single grain ages. Also note that, for reasons of clarity, only Triassic and younger dates are shown. For the complete dataset, we refer to the Supplementary Tables.

**Fig. 5.**
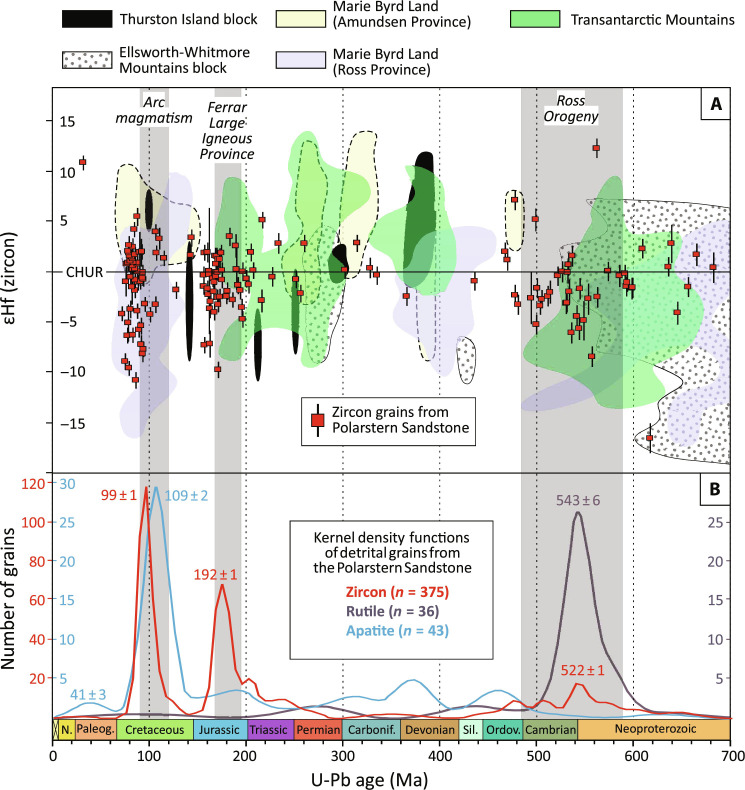
Geochronologic and isotopic record of detrital grains from the Polarstern sandstone at site PS104_20-2 compared with potential source regions and regional orogenic and magmatic events. Major orogenic and magmatic events discussed in the text are indicated by gray bars. Uncertainty bars for the isotopic data are 2SE. (**A**) U-Pb dates of detrital zircon grains, plotted against their εHf isotopic signatures. The colored fields for the isotopic compositions of the various regions are envelopes, which outline the individual εHf zircon data points published in the literature (*35*–*45*). (**B**) Kernel density functions of detrital zircon, rutile, and apatite grains. The scale for the *y* axis is color-coded (red for zircon, cyan for apatite, and dark blue for rutile).

The youngest age group contained in the Polarstern Sandstone comprises apatite and zircon grains with high εNd and εHf values of >5 and Cenozoic U-Pb and fission track dates overlapping within uncertainty limits [Fig. 4A, upper left corner; apatite fission track (AFT) average age: 38 ± 7 Ma (*n* = 2); apatite U-Pb average age: 41 ± 3 Ma (*n* = 2); zircon U-Pb age: 45 ± 2 Ma (*n* = 1)]. The AFT data contain an additional, slightly older Cenozoic age group of 53 ± 6 Ma (*n* = 5) characterized by εNd values of <5 (Fig. 4A). Other dominant populations are of middle Cretaceous age [AFT: 87 ± 5 Ma; 48% of all ages (Fig. 4, A and B); apatite U-Pb: 109 ± 2 Ma (50%); zircon U-Pb: 99 ± 1 Ma (31%) (Figs. 4A and 5, A and B)] and early Jurassic age [AFT: 190 ± 14 Ma (30%) (Fig. 4A); zircon U-Pb: 192 ± 1 Ma (33%) (Figs. 4A  and 5, A and B)]. Moreover, the rutile U-Pb dates form a well-defined Cambrian age group (543 ± 6 Ma; 81%) (Fig. 5B). A similar but more loosely defined age group is obtained from the zircon U-Pb data, clustering around a mean age of 522 ± 1 Ma (25%) (Fig. 5, A and B). The Polarstern Sandstone furthermore contains some zircon, apatite, and rutile grains with Proterozoic and/or Archean U-Pb dates (fig. S5). Zircon U-Pb dates of the rhyolitic pebbles range from ~118 to 96 Ma, those from the onshore rhyolitic exposures of the Jones Mountains cluster at 97 to 96 Ma (fig. S6).

### Biomarkers

The Polarstern Sandstone is devoid of microfossils and macrofossils that may provide biostratigraphic information. Lipid biomarker analyses, however, yielded paleoclimatic and paleoenvironmental information. We detected heterocyte glycolipids HG_26_ diols and keto-ols as well as HG_28_ diols and keto-ols, organic molecules consisting of a sugar functionality glycosidically bound to long carbon chains with hydroxy and/or ketone functionalities, which are exclusively synthesized by N_2_-fixing heterocytous cyanobacteria (*16*) that are common in freshwater. Application of the HDI_26_ (heterocyte diol index of 26 carbon atoms) lipid paleothermometer (*17*) using a previously established modern lake surface sediment calibration (*18*) yielded a reconstructed summer water temperature of 18.7°C (with a calibration error of ±1.7°C) for the depositional environment.

In addition to heterocyte glycolipids, we also detected a diverse suite of isoprenoid and branched glycerol dialkyl glycerol tetraethers (GDGTs) and their derivatives in the sandstone (fig. S7). Among these, archaeal-derived isoprenoid GDGTs were most prominent and GDGT-0 most abundant. The GDGT-0/crenarchaeol ratio was 5.2, and the branched and isoprenoid tetraether (BIT) index showed a value of 0.61 (table S12). In addition, high relative abundances of glycerol monoalkyl glycerol tetraethers (GMGT-0), frequently observed in peats (*19*), were observed. Contamination by biomarkers from the underlying late Cretaceous mudstone can be excluded because both lithologies show very different biomarker profiles; while the Eocene sandstone only contains HG_26_ diols/keto-ols and HG_28_ diols/keto-ols, the Cretaceous mudstone is characterized by the exclusive presence of HG_30_ triols/keto-diols.

## DISCUSSION

### Depositional age and environment

The youngest dates of grains contained in the Polarstern Sandstone define its maximum depositional age as middle Eocene (~44 Ma; weighted mean of the Cenozoic U-Pb dates) (fig. S8). The overlying seismostratigraphic unit was drilled at MeBo site PS104_21-3 ~40 km further north (Fig. 2) and was dated as earliest Oligocene (~34 Ma) (*20*). Hence, the Polarstern Sandstone was deposited between the middle Eocene and the latest Eocene (~44 to –34 Ma). The Polarstern Sandstone overlies a late Cretaceous mudstone dated to 93 to 83 Ma (*4*). Accordingly, the boundary between the Polarstern Sandstone and the lignite layer, which caps the mudstone and is part of the late Cretaceous sequence (*4*), represents a hiatus of at least 40 Myr (Fig. 3, A and E) resulting from either slow erosion or no deposition. This hiatus is interpreted to correlate with unconformity ASS-u1 identified in offshore seismic profiles (Fig. 2) (*21*) and is thus of regional extent. The timing of the hiatus coincides with the development of the WAES between the late Cretaceous (*5*) and the latest Paleocene to Eocene (~60 to 50 Ma) (*6*). The WAES formed as a result of very low exhumation rates and slow down-wearing after the breakup and separation of Zealandia from Antarctica and before the onset of Cenozoic tectonic activity in the West Antarctic Rift System (*8*, *22*, *23*). We suggest that both the formation of the WAES and the hiatus in our sediment core (reflected by the regional-scale seismic unconformity ASS-u1) (*21*) are associated with each other and that they are both expressions of the same tectonic regime of quiescence and relative inactivity, which prevailed in West Antarctica during the late Mesozoic and early Cenozoic.

When sediment deposition resumed in the middle to late Eocene, it was most likely in a marine-influenced, high-energy environment, such as a river delta, estuary, or coastal setting. This is indicated by sedimentological evidence, such as (i) cross-stratification and fining-upward sequences within the Polarstern Sandstone (fig. S1), (ii) high aspect ratios as well as high sphericity and convexity values of the sand grains implying fluvial or coastal grain abrasion (Fig. 3D) (*24*, *25*), and (iii) the presence of magnesium and iron carbonates, zeolites, and halites detected in the bulk mineralogy of the sandstone (*4*). The sedimentological evidence is in agreement with biogeochemical evidence, namely, the results of the BIT index, a quantitative measure for the loading of terrestrial organic matter to the oceans (*26*), which, in the Eocene sandstone, has a value of 0.61. Comparable values have been reported previously from coastal-marine environments (*26*), suggesting that the paleoenvironmental setting received a substantial contribution of riverine transported organic and siliciclastic material. The presence of high relative abundances of GMGTs (fig. S7) (*19*) and the high GDGT-0/crenarchaeol ratio may argue for the existence of extensive peat deposits in the river catchment that developed under a warm and humid climate, in agreement with the reconstructed HDI_26_ surface water temperatures (SWTs).

An Eocene river delta in today’s Amundsen Sea Embayment is also implied by seismic reflection data from the shelf between MeBo70 drill sites PS104_20-2 and PS104_21-3. These show reflectors in the late Eocene seismic unit ASS-2 that are characteristic of fluvial plains and delta systems (Fig. 2) (*27*). The reflectors include characteristics such as subparallel to chaotic structures interpreted as braided meandering river deltas or plains and imbricated prograding foresets that imply a delta front. Subsequent tectonic faulting and folding of the strata prevents a continuous mapping along the seismic profiles, but the observable patterns resemble similar examples from other paleoriver plains and deltas, such as those of the early-mid Miocene Pearl River mouth in the South China Sea (*27*).

The high kaolinite content in the clay fraction of the Polarstern Sandstone matrix (Fig. 3C) suggests intense chemical weathering in the river catchment. Kaolinite usually forms from feldspar alteration under (sub)tropical conditions (*28*). Such climate conditions, however, are rather unlikely as they oppose previous climate reconstructions for the middle to late Eocene [e.g., (*29*)], as well as our HDI_26_-reconstructed summer SWT of ~19°C. Recycling of kaolinite due to the erosion of previously weathered sedimentary rocks or strata, as suggested for similarly high kaolinite contents in late Eocene sediments from Prydz Bay off East Antarctica (*30*), also seems unlikely, as—at least at the studied drill site—the underlying late Cretaceous strata are “sealed” by indurated lignite. In addition, feldspar in the rhyolite pebbles from the Polarstern Sandstone is strongly altered. The potential source rocks of the rhyolitic pebbles exposed in the Jones Mountains, in contrast, show low kaolinite contents and largely unaltered feldspar crystals (fig. S3 and Supplementary Tables) arguing against erosion of an already weathered source.

Alternatively, we suggest that kaolinite formed during diagenetic feldspar degradation due to highly acidic meteoritic and pore waters. This process was previously described as *Moorverwitterung* (*31*) and is supported by (i) low feldspar to quartz ratios in the sand fraction; (ii) occurrence of Fe(Ca) carbonates (siderite and ankerite) (*32*) in the Polarstern Sandstone; (iii) high percentage of detrital apatite grains, which show naturally formed etching features (fig. S2); and (iv) biomarkers contained in the Polarstern Sandstone, which suggest peat deposits in the river catchment. Kaolinite formation by the process of *Moorverwitterung* requires the presence of humic acids produced in a swamp environment (*31*). We suggest that the Polarstern Sandstone was originally overlain by or intercalated with humus-rich layers, from which acidic water percolated into the sandstone as groundwater. These humic layer(s) may have been later removed by erosion, i.e., fluvial erosion since the late Eocene and/or subglacial erosion from repeated subsequent ice sheet advances (*7*).

### Early volcanism and a >1500-km-long transcontinental West Antarctic river system

To constrain the course and the size of the river system that deposited the Polarstern Sandstone, we undertook provenance analysis of detrital grains and pebbles using isotope geochemistry and geo/thermochronology under the assumption that age distributions and geochemical characteristics of the detritus reflect those of the river’s source areas.

The sandstone contains detrital grains of a small but distinct age group characterized by Cenozoic AFT and apatite and zircon U-Pb dates (figs. S8 and S9) with juvenile Hf and Nd isotopic signatures (Fig. 4). The ɛNd values of these young apatites are indistinguishable at the 1 σ level from those of volcanoes exposed today in Marie Byrd Land and the Thurston Island blocks of West Antarctica (Fig. 1), forming one of the world’s largest and still active volcanic provinces (*12*, *33*). The onset of the main period of rift-related volcanism in West Antarctica was previously dated at ~30 to 28 Ma (*10*, *11*). The only earlier volcanic activity of the Marie Byrd Land volcanic province was reported from Mt. Petras (Fig. 1), which may have started as early as 36 Ma (*10*). Our U-Pb dates suggest an even earlier onset of volcanic activity at ~44 Ma. This age agrees with the age of oldest rift-related magmatism observed from North Victoria Land in the northernmost Transantarctic Mountains, ~2500 km away from the Amundsen Sea Embayment (Fig. 1). Here, alkaline dykes were dated as 45 to 44 Ma (*34*), showing that early rift-related magmatism of middle to late Eocene age affected different areas of Antarctica and was hence more widespread than previously thought.

The Polarstern Sandstone contains abundant rhyolitic pebbles. These, together with ~30 to 50% of the detrital zircon and apatite grains, yield Cretaceous U-Pb and fission track ages of ~110 to 90 Ma (Figs. 4 and 5 and Supplementary Tables). These ages as well as the isotopic and trace element compositions of detrital zircon and apatite are typical of arc magmatic rocks (Fig. 5 and fig. S5) (*35*–*38*), which formed when Zealandia was still connected to Antarctica and the proto-Pacific Ocean was subducting beneath this continental land mass. Today, these Cretaceous magmatic rocks dominate the coastal exposures of Marie Byrd Land and the Thurston Island Block (Fig. 1) and are thus the most likely source of the Cretaceous age groups contained in the sandstone. An origin from the Thurston Island Block is also most likely for the rhyolitic pebbles, whose Cretaceous zircon U-Pb dates overlap with those of the rhyolitic exposures of the Jones Mountains (Thurston Island Block; Fig. 1 and fig. S6) (*15*). In contrast, rocks with Cretaceous U-Pb ages are only subordinately exposed in the Ellsworth-Whitmore Mountains and the Transantarctic Mountains (Fig. 1) as these areas were situated outside the magmatic arc during Mesozoic mountain building. Hence, these areas are unlikely to have contributed to the Cretaceous U-Pb age groups. In conclusion, the Cretaceous U-Pb age signal indicates sediment discharge from a proximal, West Antarctic source, that is, the Marie Byrd Land and Thurston Island crustal blocks situated along the coast of the Amundsen Sea.

**Fig. 6.**
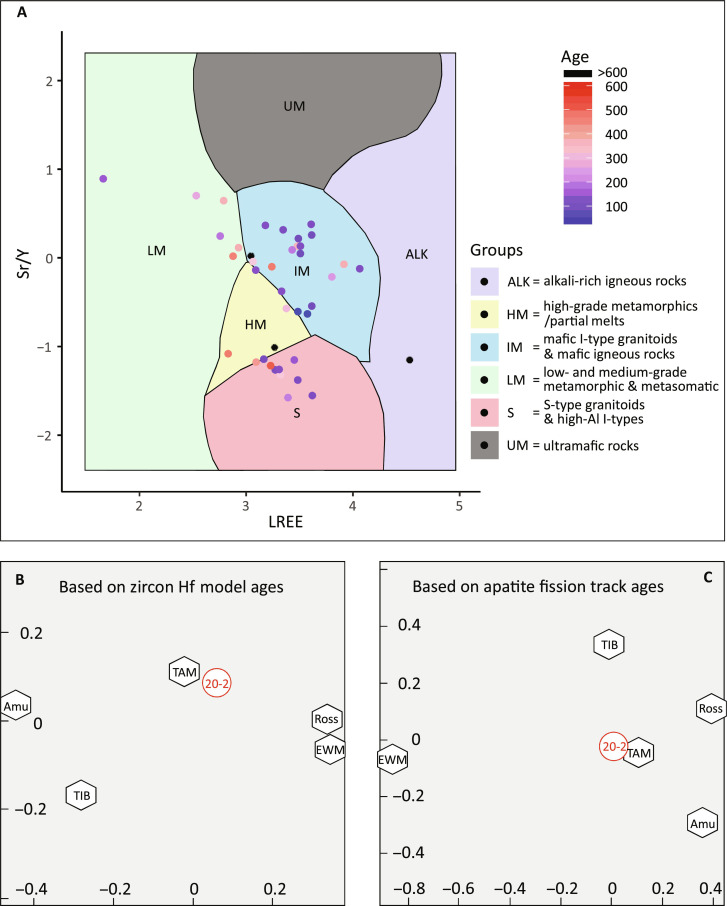
Comparison of data from the Eocene Polarstern Sandstone with different source areas and rock types. (**A**) Detrital apatite from the Polarstern Sandstone plotted on Sr/Y versus LREE support vector machine biplot with color-coded U-Pb ages (*47*). The lithological fields are derived from a bedrock apatite reference library (*47*). Lower panel: Multidimensional scaling (MDS) plots showing the congruence of detrital data from the Polarstern Sandstone of this study (20-2) with data from potential source (*6*, *8*, *14*, *22*, *23*, *35*–*45*, *49*–*62*, *79*, *80*). (**B**) MDS plot based on zircon Hf model ages. (**C**) MDS plot based on AFT ages. Abbreviations: Amu, Amundsen Province of Marie Byrd Land (see Fig. 1); EWM, Ellsworth-Whitmore Mountains block; Ross, Ross Province of Marie Byrd Land (see Fig. 1); TAM, Transantarctic Mountains; TIB, Thurston Island block.

Evidence for input from more distal sources is provided by zircon, apatite, and rutile grains yielding late Proterozoic to Cambrian and Jurassic U-Pb ages (Fig. 5 and fig. S5). These ages, together with ɛHf isotopic signatures of zircons, match with those of rocks formed during the Ross Orogeny and from the Ferrar Large Igneous Province, respectively (*39*–*42*). These rocks are all widely exposed in the Transantarctic Mountains but not to such an extent on the other crustal blocks of West Antarctica (Fig. 1) (*40*–*45*). In theory, debris from erosion of the Ross Orogen may also be present in the Paleozoic clastic sedimentary strata forming the host rocks into which the magmatic bodies of West Antarctica were emplaced during the Mesozoic (Swanson Formation) (*46*). The Swanson Formation has experienced a low-grade metamorphic overprint (*46*), which is known to alter the trace element geochemistry of apatite (*47*). Trace element patterns of most detrital apatite contained in the Polarstern Sandstone, however, suggest derivation from igneous sources, arguing against recycling in West Antarctic metasediments (Fig. 6A).

Multidimensional scaling plots (*48*) constructed using zircon Hf model ages and AFT ages confirm the similarity of detrital zircon/apatite and the Transantarctic Mountains as a major source (Fig. 6, B and C). Furthermore, the Polarstern Sandstone also yields Triassic-Jurassic AFT ages (Fig. 4). These ages occur commonly in the Transantarctic Mountains but rarely in West Antarctica (Fig. 4) (*6*, *8*, *14*, *22*, *49*–*62*). A Transantarctic Mountain provenance is also indicated by the occurrence of lithic arkose pebbles in the sandstone (fig. S4), which reveal notable similarities with rocks exposed in the Kukri Hills of the Transantarctic Mountains (Fig. 1) as they contain hematite-cemented grains and a high proportion of angular to subrounded quartz grains (*63*). In summary, the U-Pb dates characteristic for the Ross Orogeny and the Ferrar Large Igneous Province, the pre-Cretaceous fission track dates, and the hematite-cemented lithic arkose pebbles strongly argue for an additional, distal source of the river, situated in the Transantarctic Mountains of East Antarctica.

The Transantarctic Mountains were situated >1500 km away from the Amundsen Sea drill site during the middle to late Eocene, even when taking into account the ~180 km of extension proposed for the West Antarctic Rift System during the Cenozoic (*9*). The provenance data thus indicate a large transcontinental river system that transported sediment from the young and rising Transantarctic Mountains all the way across West Antarctica toward the South Pacific Ocean (Fig. 1).

### Tectonomorphic evolution and implications for ice sheet development

**Fig. 7.**
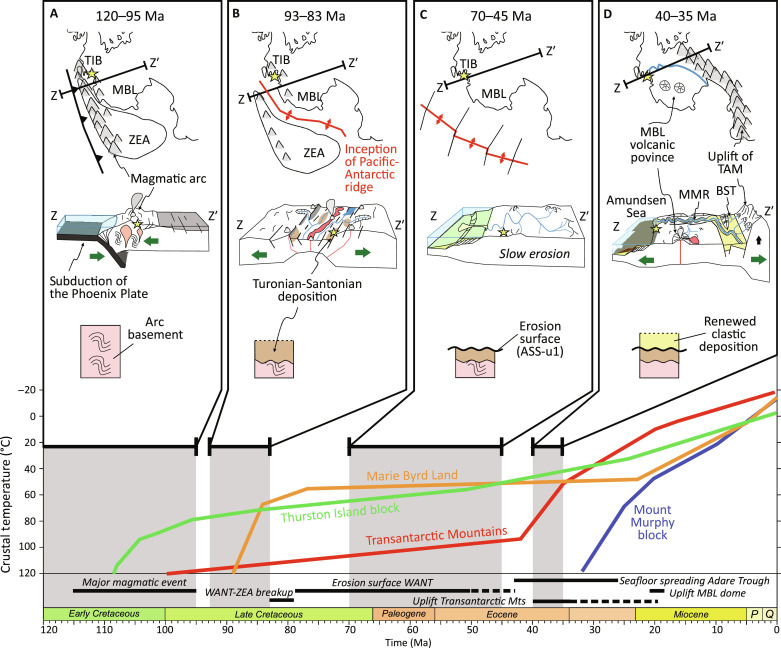
Cretaceous-Cenozoic tectonothermal and landscape evolution of West Antarctica. Upper panels show the schematic paleogeographic setting, a geological profile (Z to Z′), and a simplified evolving stratigraphic section of the uppermost crust, with the drill site PS104_20-2 (yellow star) for the individual time slices. The lower diagram shows representative thermal histories from relevant geological areas (*8*, *14*, *23*). (**A**) Early Cretaceous: West Antarctica is connected to Zealandia and forms the active margin of Gondwana with ongoing subduction and mountain building. (**B**) Late Cretaceous: Subduction has ceased, West Antarctica is in a passive margin position, and the South Pacific Ocean starts to open and separates Zealandia from Antarctica. (**C**) Latest Cretaceous to early Paleogene: West Antarctica experiences relative tectonic quiescence, and the WAES(s) and the regional-scale unconformity ASS-u1 are formed. (**D**) Middle to late Eocene: The West Antarctic Rift System is active, associated with the uplift of the Transantarctic Mountains and volcanic activity. The Polarstern Sandstone is deposited by a large transcontinental river system. Abbreviations: BST, Bentley Subglacial Trench; MBL, Marie Byrd Land; MMR, Mount Murphy Rift; TAM, Transantarctic Mountains; TIB, Thurston Island block; WANT, West Antarctica; ZEA, Zealandia.

Previous thermochronology studies concluded that exhumation of the Transantarctic Mountains commenced at ~55 Ma (*54*), followed by seafloor spreading in the Adare Trough (Ross Sea; Fig. 1) initiating at ~43 Ma, and the onset of main volcanism only after this seafloor spreading ceased at ~26 Ma (*9*). Our new data, in concert with more recently published thermochronology data from the Transantarctic Mountains (*49*, *50*), suggest that (within age uncertainty limits) renewed sediment deposition in the Amundsen Sea Embayment, rift-related magmatism, seafloor spreading of the Adare Trough, and the rise of the Transantarctic Mountains all started synchronously within the same time interval from ~44 to 40 Ma (Fig. 7). Volcanism, topography formation, and renewed sedimentation hence all reflect the initiation of Cenozoic rift activity, following ~40 Myr of relative tectonic quiescence.

Our data indicate that the Eocene fluvial drainage system differed strongly from the modern, glacial drainage system. The divide of today’s Amundsen Sea drainage basin of the WAIS, which extends to the northern tip of the Ellsworth-Whitmore Mountains (Fig. 1), must have reached further toward the Ross Sea during the middle to late Eocene to allow for transport of detritus from the Transantarctic Mountains to the Amundsen Sea (Figs. 1 and 7). Accordingly, the size of the Amundsen Sea river catchment must have been much larger and differed considerably from previous assumptions of preglacial Antarctic river networks (which were based on the modern Antarctic topography) (*64*). The appearance in the Ross Sea stratigraphic record at ~24.5 Ma of zircon yielding Cretaceous to Triassic U-Pb ages characteristic of West Antarctica (*65*) provides a minimum age for capture of the distal part of this drainage system (*66*). Capture is therefore constrained between ~44 to 34 Ma (Polarstern sandstone deposition) and ~24 to 25 Ma, synchronously with uplift of the Mt. Murphy block and Marie Byrd Land dome (Fig. 7) (*8*). As the onset of continent-scale glaciation in West Antarctica is still poorly constrained, it is unclear whether that capture occurred against a background of a fluvial or a glacial drainage system. It is also unclear when the fluvial drainage from the Transantarctic Mountains to the Amundsen Sea was ultimately disrupted. The growth of the WAIS and hence the transformation to glacial conditions may have rerouted and eventually cut off the fluvial network. Alternatively, thermal subsidence of the West Antarctic Rift System may have caused the formation of a seaway within the continental interior of West Antarctica, thus cutting off the river system.

The exact location of the West Antarctic paleoriver system cannot be constrained precisely. However, it seems likely that it originated in the valley systems oriented perpendicular to the Transantarctic Mountains, whose fluvial origin has been inferred from their geomorphological record (*64*, *66*). The most likely scenario—following the structural trend of the developing rift system—is a river network that flowed parallel to the Transantarctic Mountains (Fig. 1), following a narrow basin beneath the Ross Ice Shelf, as indicated by increasing sedimentary thickness within this basin along the proposed course of the river system (*67*). A direct discharge into the Ross Sea was presumably blocked by a highland north of the present-day Ross Ice Shelf (*67*). The river system then presumably turned north toward the Amundsen Sea Embayment, captured by the north-south directed Mt. Murphy Rift (Fig. 1), which was active since at least the early Oligocene (*8*).

**Fig. 8.**
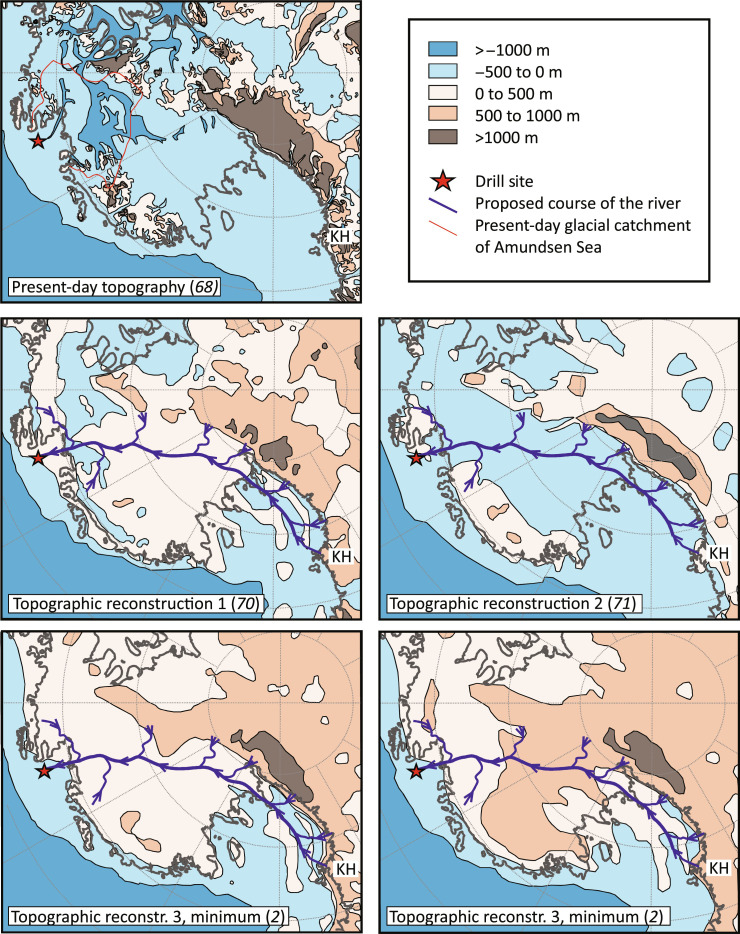
Reconstructions of the middle to late Eocene topography of West Antarctica, compared to the present day. (**Top**) Present-day subglacial topography (*68*). (**Center left**) Reconstruction of Paxman *et al.* (*70*) for the Eocene-Oligocene transition (~34 Ma). The flat-lying topography of most of the West Antarctic interior is in agreement with our proposed river course, as is the coastal position of the drill site. However, the flooded areas parallel to the present-day coast line cannot be reconciled with our data. (**Center right**) Reconstruction of Scotese and Wright (*71*) for the late Eocene (~36 Ma). The flooded interior of West Antarctica would prevent river transport from the Transantarctic Mountains to the Amundsen Sea Embayment and thus disagrees with our data. (**Bottom left**) Minimum topography model for the Eocene-Oligocene transition (~34 Ma) by Wilson *et al.* (*2*), in good agreement with our data. According to our interpretation, however, the sandstone was deposited in a river delta or estuarine environment, so the coastline should be closer to the drill site. (**Bottom right**) Maximum topography model for the Eocene-Oligocene transition (~34 Ma) by Wilson *et al.* (*2*). The ongoing formation of the WAES and the low erosion rates derived from the thermochronological data render a mountainous interior of West Antarctica unlikely. The partly emergent western Ross Sea would agree with the headwaters of the river system rooted in the Kukri Hills of the Transantarctic Mountains.

Our data have substantial implications for the Eocene West Antarctic topography, providing important boundary conditions for the onset of continental glaciation, which is still poorly constrained for West Antarctica (*2*, *8*). Today, large areas of West Antarctica (excluding the Antarctic Peninsula) lie below sea level (*68*), even after adjusting for the isostatic effect of the ice load (Fig. 8). Deep troughs, such as the Bentley Subglacial Trench (Fig. 1), reach to nearly 2.5 km below sea level (*68*). Hence, most of the WAIS is marine-based, i.e., it is grounded below sea level on the continent. During the late Eocene, when elevated regions of East Antarctica and the Antarctic Peninsula may have already started to glaciate (*69*), ocean temperatures were presumably still too warm to allow the formation of a marine-based ice sheet. Thus, the presence of topography above sea level across the center of West Antarctica is a critical prerequisite for the formation of a large-scale terrestrial ice sheet (*2*, *8*).

Recent reconstructions of Eocene topography range from a fully submerged interior of West Antarctica to a hilly topography with elevations between ~500 and 1000 m above sea level (Fig. 8) (*2*, *70*, *71*). The existence of a large river system stretching from the Transantarctic Mountains to the Amundsen Sea coast, as indicated by our data, shows that the interior of West Antarctica must have been above sea level, supporting the paleotopographic reconstruction of Paxman *et al.* (*70*) and the minimum topography model of Wilson *et al.* (*2*) (Fig. 8). It also implies a topographic gradient between the young Transantarctic Mountains and the Amundsen Sea Embayment. The occurrence of hematite-bound lithic arkose pebbles in the Polarstern Sandstone suggests that the headwaters of the river system were situated in the Kukri Hills of the Transantarctic Mountains. This would imply that, in addition to the West Antarctic interior, parts of the present-day Ross Sea were also above sea level, as suggested by the maximum topography model of Wilson *et al.* (*2*). This part of our proposed river course, however, is associated with high uncertainties as we cannot exclude that similar hematite-bound lithic arkose lithologies were also exposed in other parts of the Transantarctic Mountains, outside of the Kukri Hills. Very low West Antarctic erosion rates during the early Cenozoic (*8*, *22*, *23*), together with the formation of an erosion surface (*5*) and the long depositional hiatus documented in our drill core, suggest little relief and a subdued topography close to sea level. Moreover, some areas in interior West Antarctica must have been located below sea level between ~47 and 45 Ma (i.e., shortly before the deposition of the sandstone), as is evident from recent findings of reworked marine microfossils in subglacial tills underlying modern ice streams along the Siple Coast (*72*).

Our new data indicate that over ~40 Myr of relative tectonic quiescence, West Antarctica became a vast, flat, and low-lying plain, characterized by very slow erosion inland associated with a regional-scale hiatus in the coastal and offshore sedimentary record. Renewed tectonic activity in the middle to late Eocene caused the onset of volcanic activity, contributed to the rise of the Transantarctic Mountains, and the establishment of a major transcontinental river system extending from the Transantarctic Mountains toward the Pacific Ocean coast. Despite the existence of a major East Antarctic ice sheet, which initiated in the rising Transantarctic Mountains at the Eocene-Oligocene boundary (*69*), extensive ice sheet cover on top of largely low-lying West Antarctica was unlikely.

## MATERIALS AND METHODS

### Zircon, rutile, and apatite sample preparation

Detrital samples were collected from different depths of MeBo drill core PS104_20-2 (table S2). These samples comprised semilithified sands and lithic clasts/pebbles. The semilithified sands were crushed with a pestle to disaggregate the sample and were wet sieved using 63- and 315-μm sieves. The 63- to 315-μm fraction was then subjected to magnetic and heavy liquid (lithium heteropolytungstate and diiodomethane) separations to obtain enriched zircon, rutile, and apatite separates. The respective minerals were handpicked for final grain selection under an Olympus SZ61 binocular microscope. Virtually, no sample bias was introduced because, in most cases, all respective mineral grains were selected for mount preparation due to the generally very small amount of sample material. Larger lithic (rhyolite) pebbles sampled from the MeBo drill core (table S2) were cleaned and then crushed with a pestle. The nonmagnetic 63- to 315-μm zircon fraction was obtained by the same protocol as for the detrital samples described above. Basement samples from the Jones Mountains were cleaned, crushed with a jaw crusher, and then processed as previously described to obtain enriched zircon separates. For all analyses, grains were mounted in epoxy resin, ground to reveal internal surfaces, and polished.

### U-Pb analyses

All U-Pb analyses were carried out using a Photon Machines Analyte Excite 193-nm ArF excimer laser-ablation system with a HelEx two-volume ablation cell coupled to an Agilent 7900 ICPMS at the Department of Geology, Trinity College Dublin. The laser fluence was set at 2.5 J/cm^2^ with a repetition rate of 15 Hz and analysis time of 20 s, followed by an 8-s pause to allow for signal washout and a subsequent baseline measurement. Spot sizes of 47 and 24 μm were used for apatite and zircon, respectively, in separate analytical sessions.

Data reduction used the Vizual_Age and VisualAge_UComPbine data reduction schemes (DRSs) for Iolite for zircon and apatite, respectively (tables S3 to S5 and S7). Each DRS corrects for intrasession analytical drift, mass bias, and downhole fractionation using a user-specified fractionation model based on measurements of the primary standard; additionally, VisualAge_UComPbine permits the presence of a variable common Pb (Pb_c_) content in a primary age standard to be corrected for using a known initial ^207^Pb_c_/^206^Pb_c_ value.

The generally concordant behavior of the U-Pb system in zircon allowed data filtering based on discordance. Single-grain analysis concordia ages were calculated, and analyses with a probability of concordance of <0.001 were rejected for detrital datasets (tables S3 to S5). The primary standard was Plešovice zircon; the GZ7, AUS2, and 91500 zircons were used as secondary standards and treated as unknowns during data reduction and age calculation (table S6).

For apatite analyses, Madagascar apatite was used as the primary standard and the McClure Mountain and Durango apatites were used as secondary standards (table S6). Unlike phases that exclude Pb_c_ during crystallization, apatite grains are typically discordant in the U-Pb isotopic system. Pb_c_ in the secondary standards was corrected for using fixed initial ratios, yielding weighted mean ages of 532.2 ± 6.0 and 32.3 ± 0.7 Ma. Variable common Pb contents in the detrital apatite unknowns were corrected by using the terrestrial Pb evolution model of Stacey and Kramers (table S7) for calculation of single-grain ages. This model provided a starting estimate for ^207^Pb_c_/^206^Pb_c_ values to which a 1% uncertainty was assigned, followed by an iterative calculation to obtain single-analysis ^207^Pb-corrected ages (table S7).

The procedures used in rutile U-Pb analysis were similar to those described for apatite U-Pb, except that the primary standard was the R10 rutile and the secondary standards were the R19, PCA, and SUG rutiles for ages obtained on secondary standards (table S6). As with apatite, rutile can contain appreciable common Pb. Thus, the “Vizual-Age_UcomPbine” DRS was used with a ^207^Pb-based correction (table S8).

For trace element analysis of apatite, NIST612 was used as the primary standard, and Durango apatite was used as the secondary standard (table S7). The trace element DRS for Iolite was used to correct for sample-standard differences in ablation yield by normalization to a stoichiometric mass (^43^Ca; table S7).

### Zircon Hf and apatite Nd isotope analyses

All isotope analyses were carried out at the NERC Isotope Geoscience Laboratory in Keyworth, United Kingdom, using a Thermo Scientific Neptune Plus MC-ICPMS (multicollector inductively coupled plasma mass spectrometer) coupled to a New Wave Research UP193UC Excimer laser ablation system and low-volume ablation cell. Helium was used as the carrier gas through the ablation cell with Ar make-up gas being connected via a T piece and sourced from a Cetac Aridus II desolvating nebulizer. Nitrogen (0.006 liter/min) was introduced via the nebulizer in addition to Ar to minimize oxide formation.

For hafnium (Hf) isotope analysis, ^172^Yb, ^173^Yb, ^175^Lu, ^176^Lu+^176^Yb+^176^Hf, ^177^Hf, ^178^Hf, ^179^Hf, and ^180^Hf were measured simultaneously during static 30-s ablation analyses. The spot size used was 35 μm; the fluence was 7 to 10 J/cm^2^. Hf reference solution JMC475 was analyzed during the analytical session, and sample ^176^Hf/^177^Hf ratios are reported relative to a value of 0.282160 for this standard. Correction for ^176^Yb on the ^176^Hf peak was made using reverse-mass-bias correction of the ^176^Yb/^173^Yb ratio empirically derived using Hf mass bias-corrected Yb-doped JMC475 solutions (table S3). ^176^Lu interference on the ^176^Hf peak was corrected by using the measured ^175^Lu and assuming ^176^Lu/^175^Lu = 0.02653. At least two zircon reference materials (91500, Mud Tank and, on occasion, Plešovice and Zr144 standard glass) were analyzed throughout the analytical session. The 91500 zircon reference material was used to normalize the ^176^Lu/^177^Hf ratio assuming a value of 0.000311 (table S9). Analytical uncertainties for unknowns were propagated by quadratic addition to include the standard error of the mean of the analysis and the reproducibility of the 91500 reference material. εHf values were calculated using a ^176^Lu decay constant of 1.867 × 10^−11^ year^−1^, the present-day chondritic ^176^Lu/^177^Hf ratio of 0.0336, and the ^176^Hf/^177^Hf ratio of 0.282785 (table S3).

For neodymium (Nd) isotope analysis, ^142^Ce, ^142^Nd, ^143^Nd, ^144^Nd+^144^Sm, ^145^Nd, ^146^Nd, ^147^Sm, ^149^Sm, ^150^Nd, and ^151^Eu were measured simultaneously during static 30-s ablation analyses. The spot size used was 35 μm; the fluence was 7 to 10 J/cm^2^. Nd reference solution JNd-i was analyzed during the analytical session, and sample ^143^Nd/^144^Nd ratios are reported relative to a value of 0.512115 for this standard. Correction for ^144^Sm on the ^144^Nd peak was carried out as described in table S7. Durango and Madagascar apatite reference materials were analyzed through the analytical session, along with the standard glasses JNd-i, JNd-i LREE, and NIST 610. Analytical uncertainties for unknowns were propagated by quadratic addition to include the standard error of the mean of the analysis and the reproducibility of the Durango reference material. εNd values were calculated using a ^147^Sm decay constant of 6.54 × 10^−12^ year^−1^, the present-day chondritic ^147^Sm/^144^Nd ratio of 0.1967, and the ^147^Sm/^144^Nd ratio of 0.512638 (table S7). Last, Lu-Hf and Sm-Nd isotope data were processed using the Iolite data reduction package (table S7).

### Fission track analysis

Spontaneous tracks were revealed by etching the grain mounts, using 5.0 M HNO_3_ at 20°C for 20 s (table S10). Grain selection and counting of spontaneous tracks were carried out with a Zeiss Axioplan microscope at ×1000 magnification at the University of Bremen. Uranium concentration measurements were performed by laser ablation inductively coupled plasma mass spectrometry (LA-ICPMS) at the Trinity College Dublin (table S10). Apatite Cl concentration measurements were obtained from sample LA-ICPMS spot ablations (table S10). Counted shards of a Durango apatite crystal were used as the LA-ICPMS fission track zeta calibration standard, while NIST612 U-doped glass was used to correct for any session drift in U/Ca ratios. The results of single-grain AFT analysis are reported in table S10.

### Point count analyses

A rounded sandstone clast (~1 cm in diameter) in the Polarstern Sandstone (23.02 to 23.05 mbsf) was cut in half for thin-section preparation. A representative thin-section image was then analyzed in the point counting software program JMicroVision (fig. S4) to quantitatively determine the modal proportions of quartz, feldspar, rock fragments, and groundmass. The program randomly selected 300 points on the image, which were assigned to their respective modal components. The results were 21.3% quartz, 11.3% feldspar, 9.6% rock fragments, and 57.8% groundmass. The quartz, feldspar, and rock fragment (QFR) modal proportions were converted to 100% and plotted on a QFR diagram (fig. S4B).

### XRD pattern analysis

X-ray diffraction (XRD) analysis was undertaken in the Central Laboratory for Crystallography of University of Bremen (table S11). The sample material (~3 g) was pulverized and homogenized in preparation for analysis. The x-ray diffractograms have been measured on a Philips X’Pert Pro multipurpose diffractometer equipped with a Cu tube (k_α_ 1.541, 45 kV, 40 mA), a fixed divergence slit of ¼°, a 16-sample changer, a secondary Ni filter, and the X’Celerator detector system. The measurements were performed as a continuous scan from 3° to 65° 2θ, with a calculated step size of 0.016° 2θ (calculated time per step was 100 s; 2θ is the angle between transmitted beam and reflected beam). Mineral identification used the Philips/PANalytical software X’Pert HighScore, which, besides mineral identification, provides a semiquantitative value for each identified mineral on the basis of relative intensity ratio (RIP) values. The RIP values are calculated as the ratio of the intensity of the most intense reflex of a specific mineral phase to the intensity of the most intense reflex of pure corundum (*I*/*I*_c_). RIP values are sparse for clay minerals and can hamper the semiquantification of the mineral assemblage in clay-rich samples.

### Clay mineral analyses

An aliquot of the clay fraction (<2 μm) was used to determine the relative contents of the clay minerals smectite, illite, chlorite, and kaolinite (table S12) on texturally oriented samples using a Rigaku MiniFlex automated diffractometer system with CoKα radiation (30 kV, 15 mA) at the Institute for Geophysics and Geology (University of Leipzig). The clay mineral identification and quantification followed standard XRD methods (table S12).

### Biomarker extraction and analyses

A sample of the sandstone (29.7 g) collected from core 9R (drill site PS104_20-2) was lyophilized for 24 hours and homogenized using a solvent-cleaned pestle and mortar. The sample was subsequently extracted using a modified Bligh and Dyer technique as described previously (*16*). An aliquot of the lipid extract was dissolved in *n*-hexane:2-propanol:H_2_O (72:27:1, v:v:v) to a concentration of 8 mg/ml, filtered through a regenerated cellulose filter (0.45-μm pore size), and subjected to high-performance liquid chromatography coupled to tandem electrospray ionization mass spectrometry (HPLC-ESI/MS^2^).

Analysis of HGs were conducted using a Waters 2690 Alliance HPLC system connected to a Micromass Quattro LC triple quadrupole MS at the Institute of Geosciences (Christian Albrechts University, Kiel) following the analytical procedure of Bauersachs *et al.* (*17*). HGs were detected in multiple reaction monitoring mode recording the transitions specified in Bauersachs *et al.* (*18*) and quantified using the QuanLynx software application. The lipid paleothermometers HDI_26_ was calculated according to Bauersachs *et al.* (*17*) and converted into SWTs using a global temperature calibration (*18*). All HG-based data are provided in table S12.

A second aliquot (~2 mg) of the lipid extract was separated by Al_2_O_3_ column chromatography into apolar and polar lipid fractions using *n*-hexane:dichloromethane (9:1, v:v) and dichloromethane:methanol (1:1, v:v), respectively. The polar fraction was dissolved in a solvent mixture of *n*-hexane:2-propanol (99:1, v:v) to a concentration of 5 mg ml^−1^ and filtered through a 0.45-μm polytetrafluoroethylene filter (Macherey-Nagel, Germany) prior to analysis by HPLC-MS at Christian Albrechts University. Briefly, isoprenoid and branched GDGTs were eluted at 30°C using a Waters Alliance 2695 HPLC system fitted with a Prevail Cyano column (2.1 × 150 mm, 3 μm; Hichrom, United Kingdom) and a guard column of the same material. The gradient program of Hopmans *et al.* (*26*) was applied. Detection of GDGTs was achieved using a Micromass ZQ single quadrupole mass spectrometer equipped with an atmospheric pressure chemical ionization interface operated in positive ion mode. GDGTs were recorded by selected ion monitoring of their [M + H]^+^ ions (dwell time = 200 ms) and quantified by integration of peak areas using the QuanLynx integration software of MassLynx. The BIT index was calculated following Hopmans *et al.* (*26*).

### Computed tomography analyses

Whole rounds of MeBo core PS104_20-2 were scanned by a Toshiba Aquilion 64 CT at the hospital Klinikum Bremen-Mitte, with an x-ray source voltage of 120 kV and a current of 600 mA and a physical resolution of 0.351 mm in *x* and *y* directions and 0.5-mm resolution in the *z* direction. Images were reconstructed using Toshiba’s patented helical cone beam reconstruction technique with a scaled resolution of 0.195 x 0.195 x 0.3 mm. CT data processing was performed with the ZIB (Zuse Institute Berlin) edition of the Amira software (version 2017.39) (fig. S1). Within Amira, core liners, including about 2 mm of the core rims, were removed from the dataset until all marginal artefacts from the coring process were removed. Subsequently, all clasts of >~1 mm, root traces (where present), and matrix sediment were segmented with the (marker-based) watershed tool of the Segmentation Editor. Markers were predominantly set by thresholding. Only in very rare cases, where the x-ray attenuation intensity differences between clasts and matrix sediment were too small for a reliable marker segmentation by thresholding (e.g., mud clasts), clast markers were segmented by hand. Holes within segmented clasts were added to the clasts with the selection fill tool. Individual clasts were separated by running a ContourTreeSegmentation (threshold: 0; persistence value: 0.05; persistence mode: relative) on the distance map of the previously segmented clasts and subsequently parameterized with the Shape Analysis module. The determined clast length was further used for a clast size analysis. Therefore, every clast within an interval of 33 CT slices (corresponds to a ~1-cm core interval) was considered, and the obtained result was written to the central slice position (unit: volume % of all segmented clasts). The analyzing interval was moved slice by slice.

## Supplementary Materials

### This PDF file includes:

Table S1: Science Team of Expedition PS104 Figs. S1 to S9 Legends for tables S2 to S17 References

### Other Supplementary Material for this manuscript includes the following:

Tables S2 to S17

Correction (3 September 2024):
Two members of the Science Team of Expedition PS104 requested their names be removed from the list in table S1. The Supplementary Materials PDF and XML have been updated. The original version is available here:
