# Supplementary material for: A large-scale transcontinental river system crossed West Antarctica during the Eocene
Source: Sci Adv. 2024 Jun 5;10(23):eadn6056. doi: 10.1126/sciadv.adn6056 (PMC11152126; doi:10.1126/sciadv.adn6056)
Supplement: Supplementary file 1 — Table S1: Science Team of Expedition PS104 Figs. S1 to S9 Legends for tables S2 to S17 References [file sciadv.adn6056_sm.v2.pdf]

CORRECTION POSTED 3 SEPTEMBER 2024

Science Advances
AAAS

Supplementary Materials for

A large-scale transcontinental river system crossed West Antarctica during the Eocene

Maximilian Zundel *et al.*

Corresponding author: Cornelia Spiegel, cornelia.spiegel@uni-bremen.de

Sci. Adv. **10**, eadn6056 (2024)
DOI: 10.1126/sciadv.adn6056

The PDF file includes:

Table S1: Science Team of Expedition PS104
Figs. S1 to S9
Legends for tables S2 to S17
References

Other Supplementary Material for this manuscript includes the following:

Tables S2 to S17

Table S1: Science Team of Expedition PS104

Name, First Name	Affiliation
Gohl, Karsten	Alfred Wegener Institute, Helmholtz Center for Polar and Marine Research, Bremerhaven, Germany
Uenzelmann-Neben, Gabriele	Alfred Wegener Institute, Helmholtz Center for Polar and Marine Research, Bremerhaven, Germany
Hochmuth, Katharina	Alfred Wegener Institute, Helmholtz Center for Polar and Marine Research, Bremerhaven, Germany
Riefstahl, Florian	Alfred Wegener Institute, Helmholtz Center for Polar and Marine Research, Bremerhaven, Germany
Dziadek, Ricarda	Alfred Wegener Institute, Helmholtz Center for Polar and Marine Research, Bremerhaven, Germany
Gebhardt, Catalina	Alfred Wegener Institute, Helmholtz Center for Polar and Marine Research, Bremerhaven, Germany
Arndt, Jan-Erik	Alfred Wegener Institute, Helmholtz Center for Polar and Marine Research, Bremerhaven, Germany
Klages, Johann	Alfred Wegener Institute, Helmholtz Center for Polar and Marine Research, Bremerhaven, Germany
Esper, Oliver	Alfred Wegener Institute, Helmholtz Center for Polar and Marine Research, Bremerhaven, Germany
Ronge, Thomas	Alfred Wegener Institute, Helmholtz Center for Polar and Marine Research, Bremerhaven, Germany
Küssner, Kevin	Alfred Wegener Institute, Helmholtz Center for Polar and Marine Research, Bremerhaven, Germany
Kuhn, Gerhard	Alfred Wegener Institute, Helmholtz Center for Polar and Marine Research, Bremerhaven, Germany
Larter, Robert	British Antarctic Survey, Cambridge, UK
Hillenbrand, Claus-Dieter	British Antarctic Survey, Cambridge, UK
Smith, James	British Antarctic Survey, Cambridge, UK
Bickert, Torsten	University of Bremen, Germany
Pälike, Heiko	University of Bremen, Germany
Frederichs, Thomas	University of Bremen, Germany
Freudenthal, Tim	University of Bremen, Germany
Zundel, Maximilian	University of Bremen, Germany
Spiegel, Cornelia	University of Bremen, Germany
Ehrmann, Werner	University of Leipzig, Germany
Bohaty, Steve	University of Heidelberg, Germany
Simões Pereira, Patric	Imperial College London, UK

Najman, Yani	Lancaster University, UK
Scheinert, Mirko	Technical University of Dresden, Germany
Ebermann, Benjamin	Technical University of Dresden, Germany

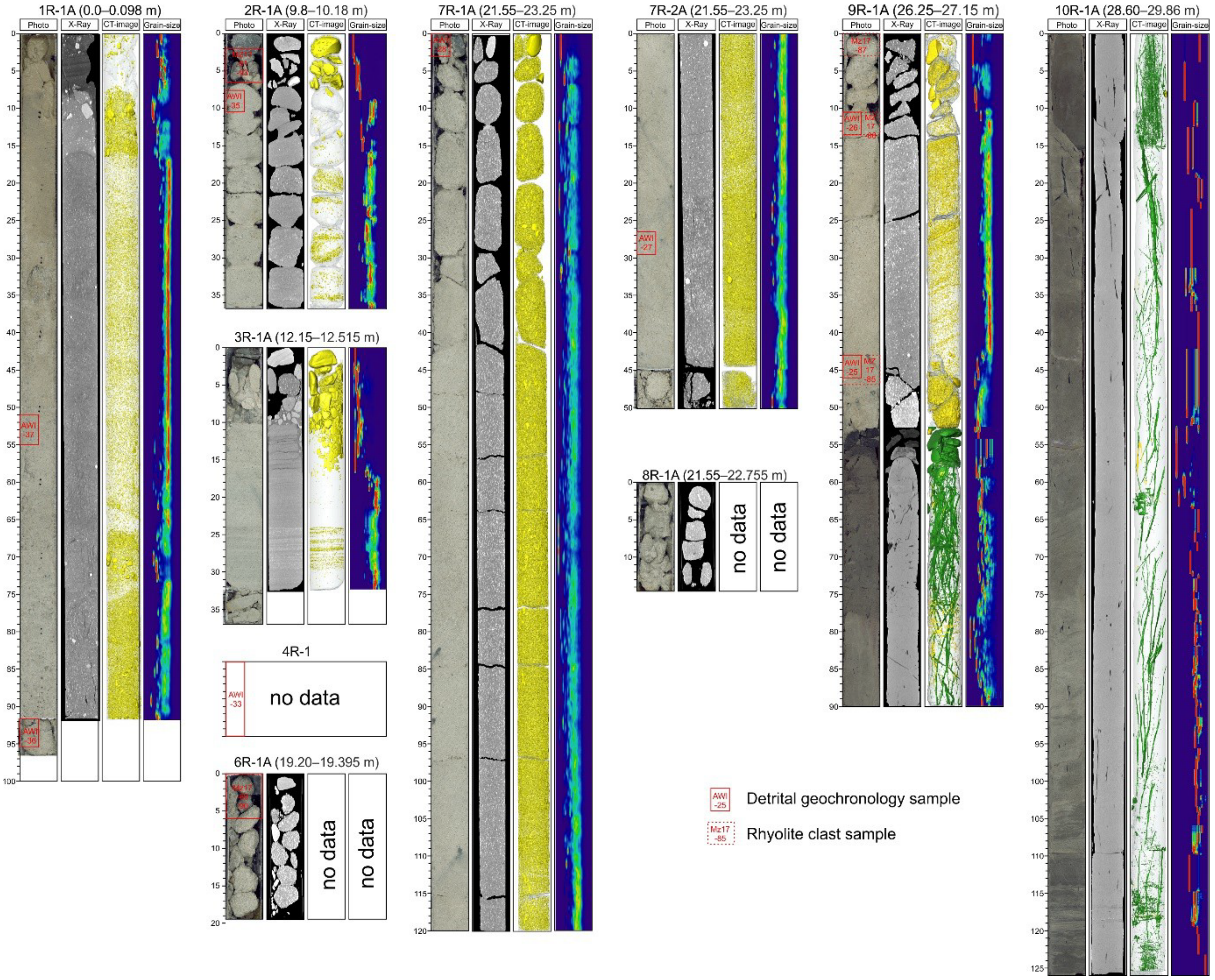

Fig. S1: Scans and grain-sized distribution from recovered core sections of site PS104_20-2. CT data processing was performed with the ZIB edition of the Amira software (version 2017.39, (114). CT-images show particles >1 mm in yellow while lignite fragments and roots are displayed in green. Grain-size distributions are scaled in phi values from -7 (left) to -1 (right), but need to be treated with care. Note that the number of clasts >1 mm below the hiatus (contained in segment 9R-1A) is very low.

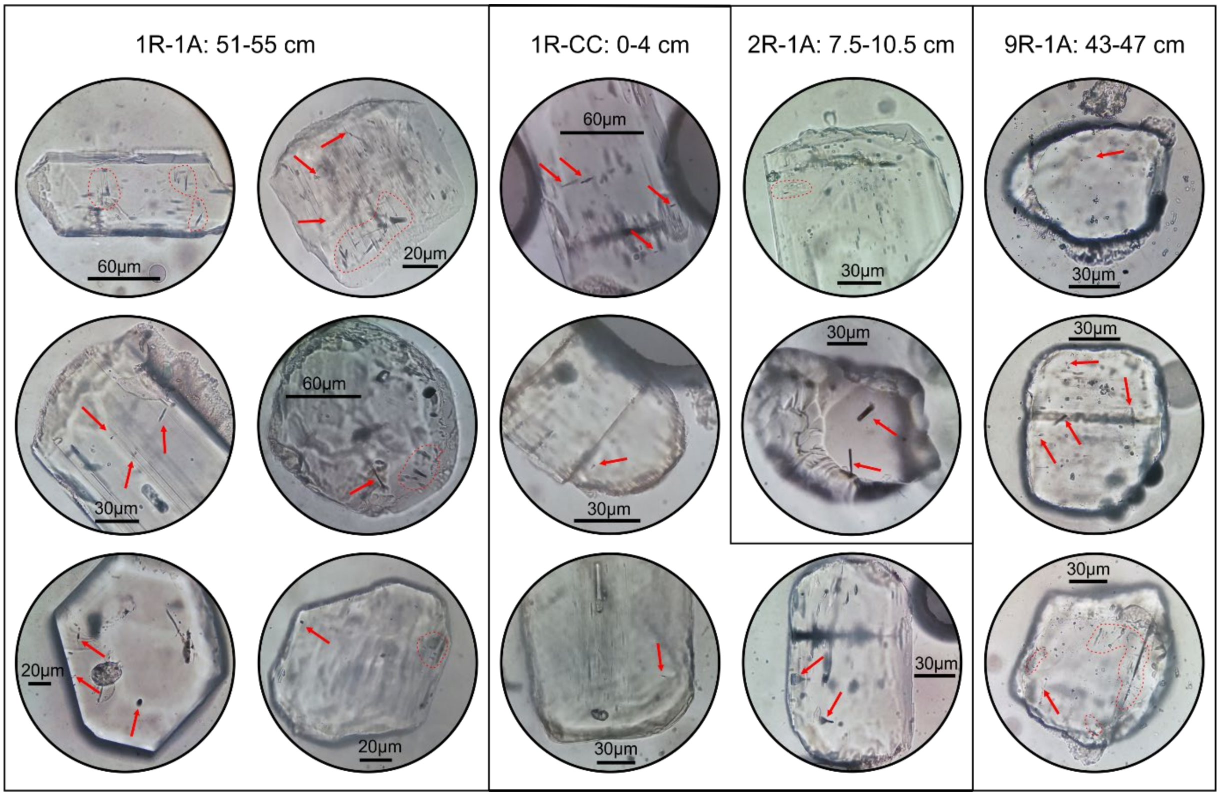

Fig. S2: Apatite crystals revealing naturally etched fission tracks and other crystal defects. Fission tracks and crystal defects are highlighted by red arrows and red dashed lines. Photomicrographs were taken of internal surfaces of apatite mounted in epoxy through an optical microscope at 1000x magnification with transmitted light.

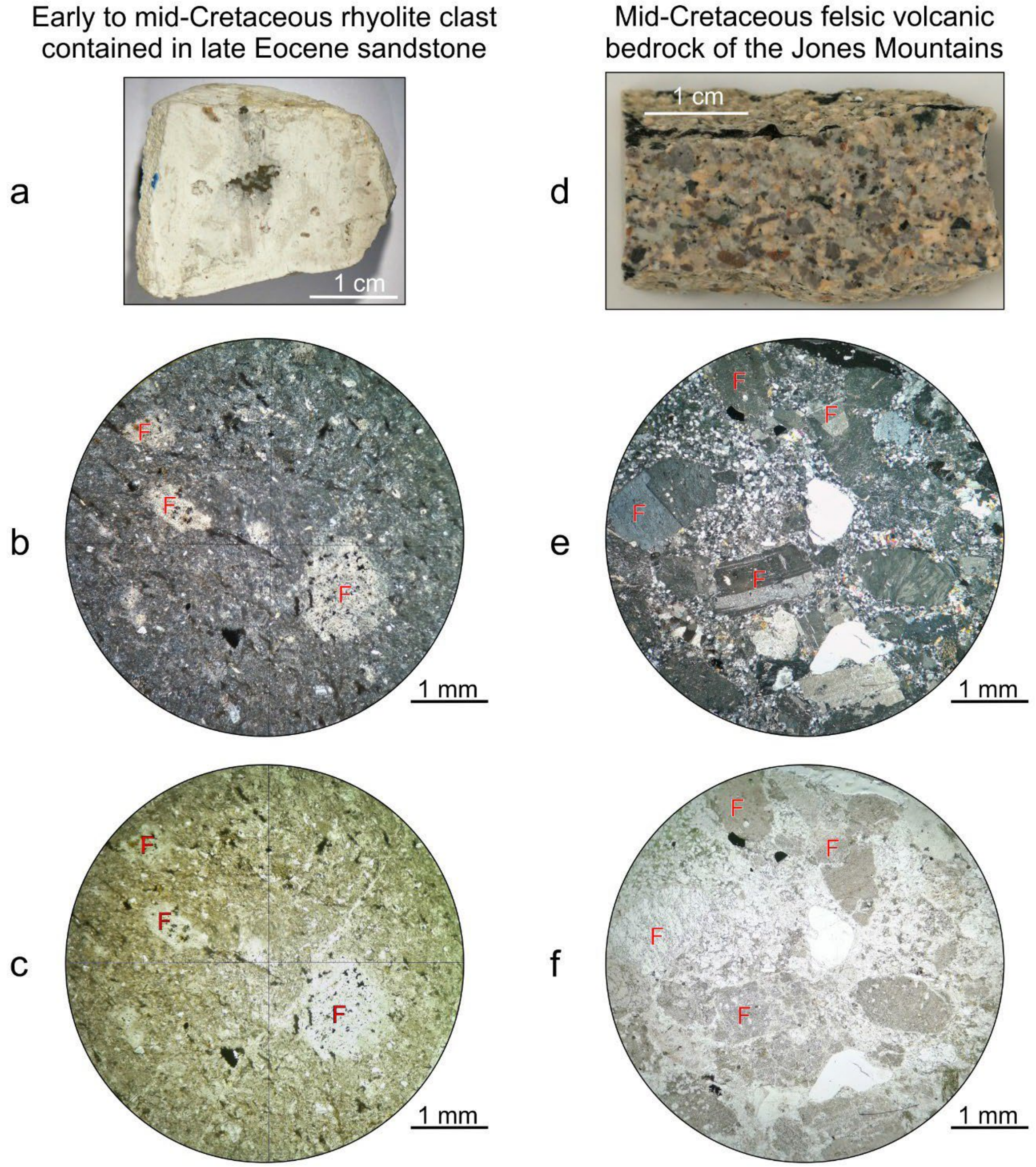

Fig. S3: Comparison of rhyolite bedrock from the Jones Mountains and rhyolite pebbles from the Polarstern Sandstone. a: Example of a rhyolite pebble contained in the Polarstern Sandstone; **b:** Thin section of the rhyolite pebble in cross-polarized light; **c**: Same thin section as (b) in plane-polarized light; **d:** Hand specimen of a rhyolitic bedrock from the Jones Mountains; **e**: Thin section of the rhyolitic bedrock in cross-polarized light; **f:** Same thin section as (e) in cross-polarized light. Note the alteration states of the feldspars (F).

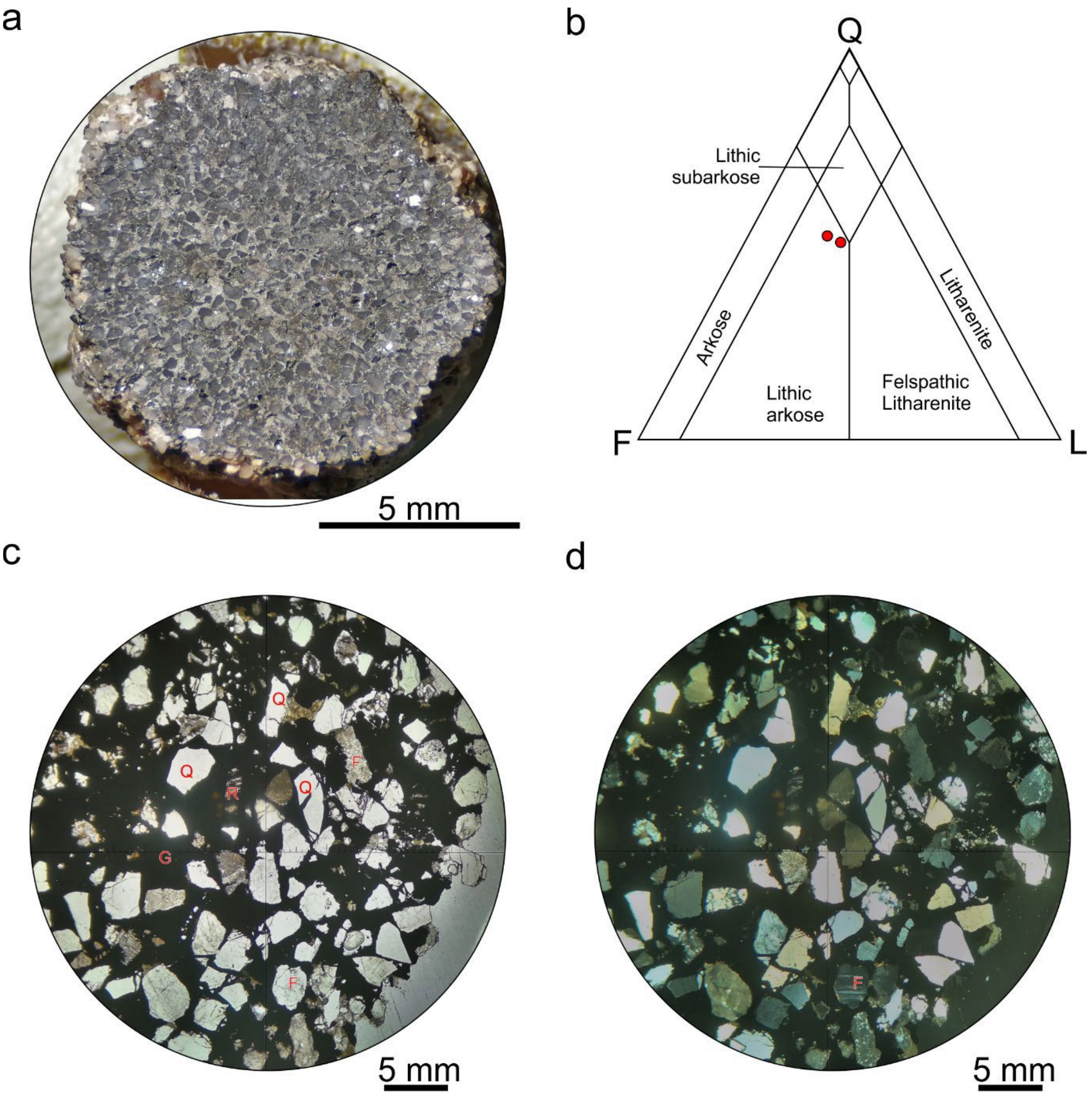

Fig. S4: Characterization of lithic arkose pebbles contained in the Polarstern Sandstone (at 26.37 and 23.04 mbsf). a: Pebble with angular grains embedded in fine-grained Fe-rich groundmass; **b**: Quartz-Feldspar-Lithic fragment (QFL)-diagram in which the sandstone pebbles (red dots) are classified as lithic arkoses based on data obtained by point-counting, using the software program JMicroVision (v1.3.1 (*108*), see Material and Methods); **c:** Plane-polarised photomicrograph showing individual components of the lithic arkose pebble. Q=quartz; F=feldspar; L=lithic clasts; G=groundmass; **d:** Corresponding cross-polarised micrograph of (c).

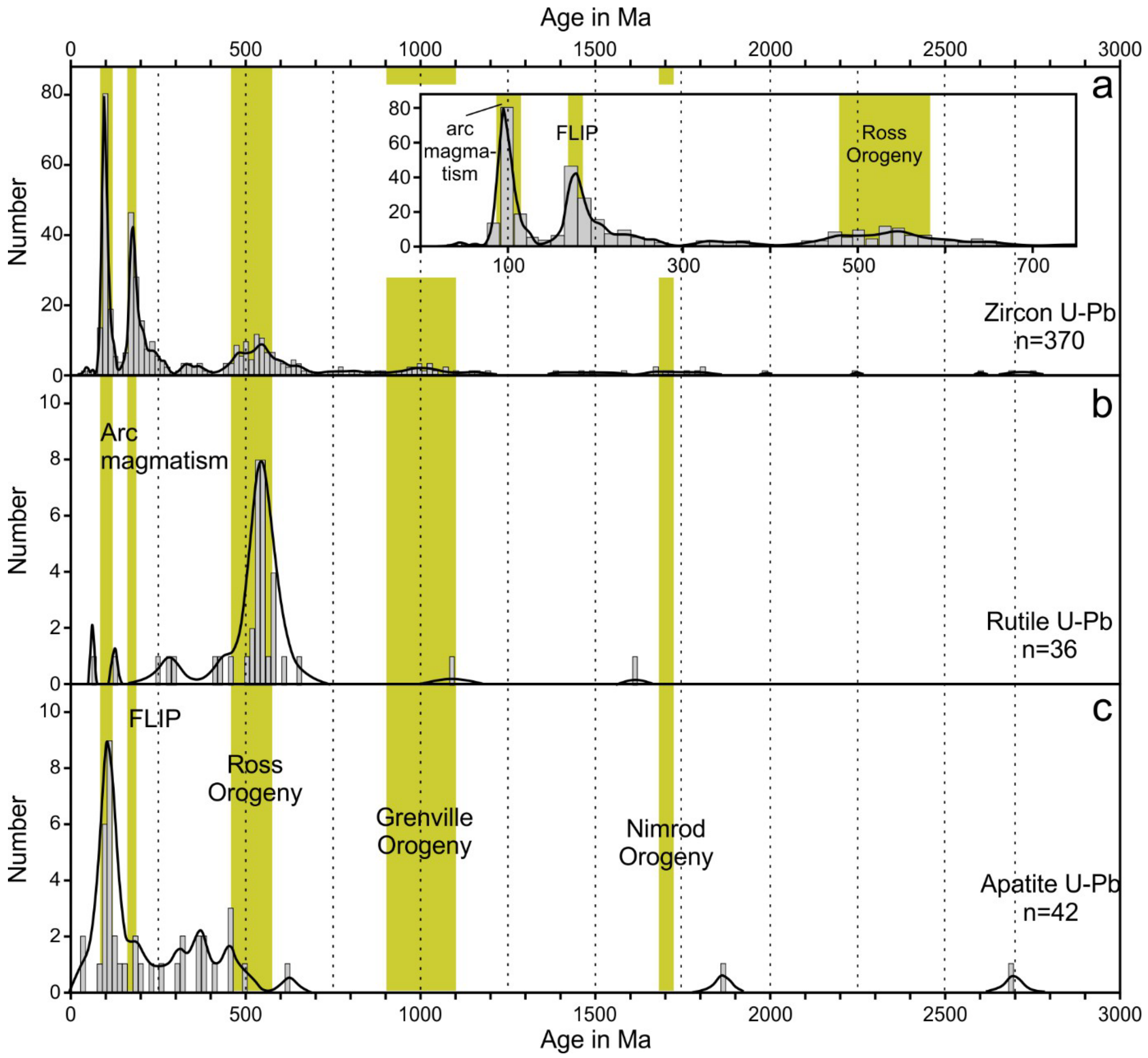

Fig. S5: Histogram and probability distribution curves of U-Pb ages. a: U-Pb ages of detrital zircon; **b:** U-Pb ages of detrital rutile; **c:** U-Pb ages of detrital apatite. Major magmatic events and orogenies are also indicated. FLIP = Ferrar Large Igneous Province.

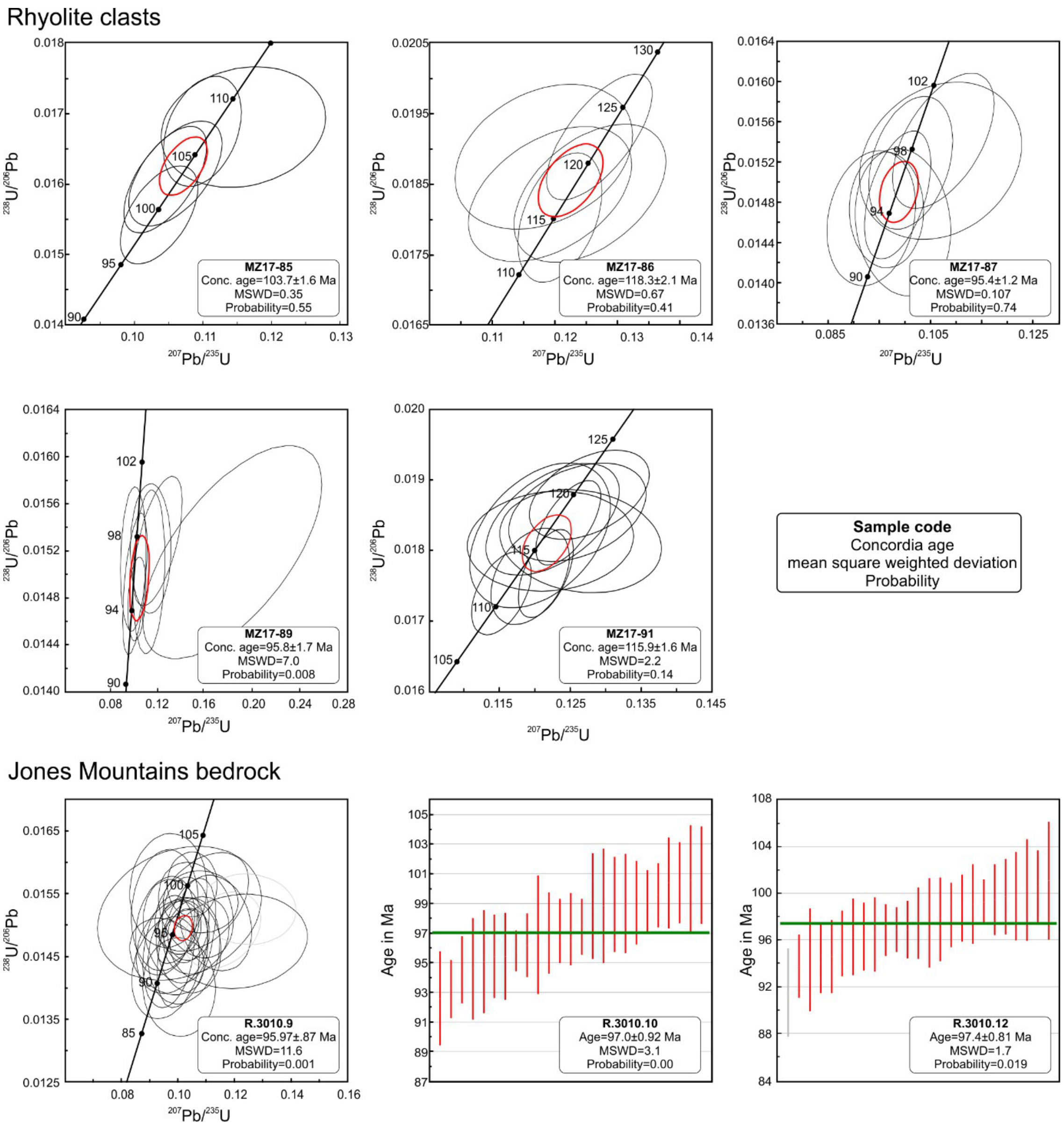

Fig. S6: Zircon U-Pb data from rhyolitic pebbles, compared to rhyolitic bedrock from the potential source area. Upper panels: Concordia plots of zircon U-Pb data for rhyolite pebbles contained in the middle to late Eocene Polarstern Sandstone. Lower panels: Bedrock volcanic and volcaniclastic rocks from the Jones Mountains. The red ellipse represents the concordia age. Note that ages displayed for samples R.3010.10 and R.3010.12 are weighted mean single-grain concordia ages.

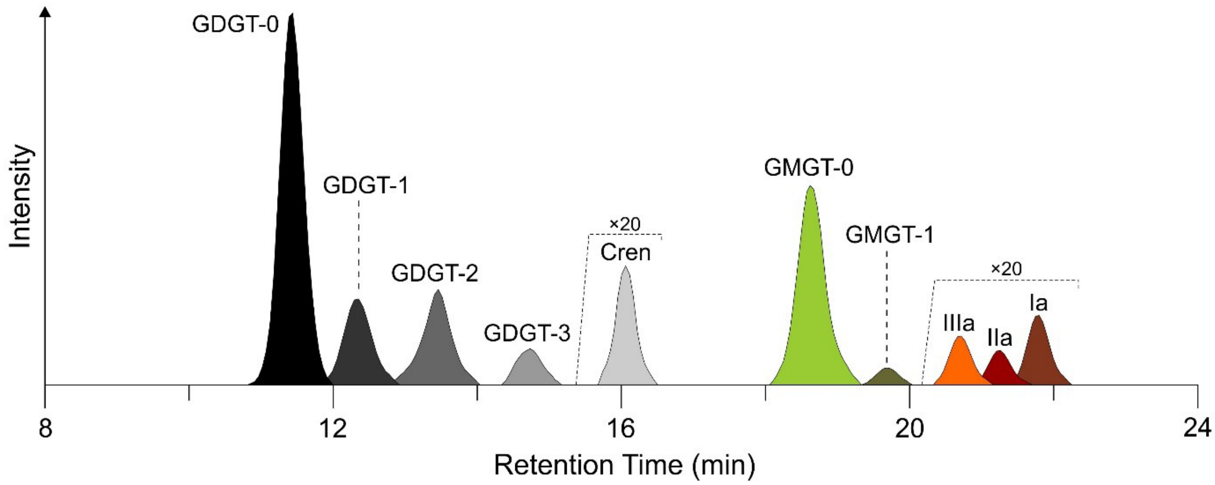

Fig. S7. Composite mass chromatogram showing the distribution of archaeal and bacterial tetraethers in the Polarstern Sandstone. Cren = crenarchaeol; GDGT= glycerol dialkyl glycerol tetraether; GMGT = glycerol monoalkyl glycerol tetraether. Numbers indicate the amount of cyclopentane rings in the molecule structure. Roman numerals refer to the different bacterial-derived GDGTs (Ia, IIa, IIIa) detected in the sandstone**.**

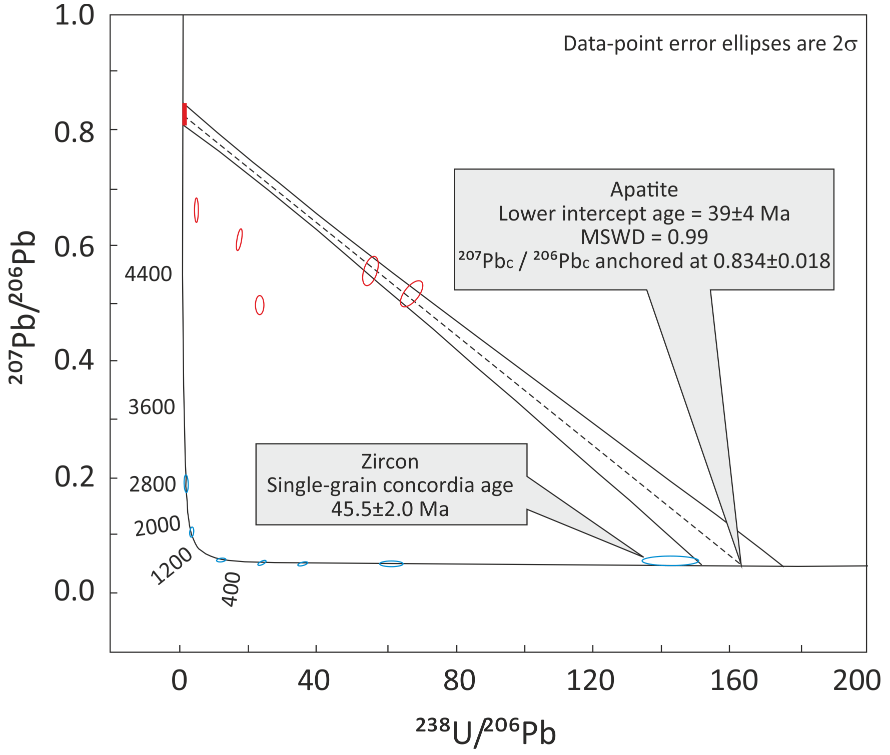

Fig. S8: Tera-Wasserburg diagram showing U-Pb data from apatite and zircon. Red symbols refer to apatite samples (9.9 mbsf); blue symbols refer to zircon samples (26.7 mbsf). Red bar at upper array intercept for Eocene apatite is the range of crystalline basement $^{207}Pb_c/^{206}Pb_c$ values for West Antarctica (*47*), which anchor the apatite age calculation.

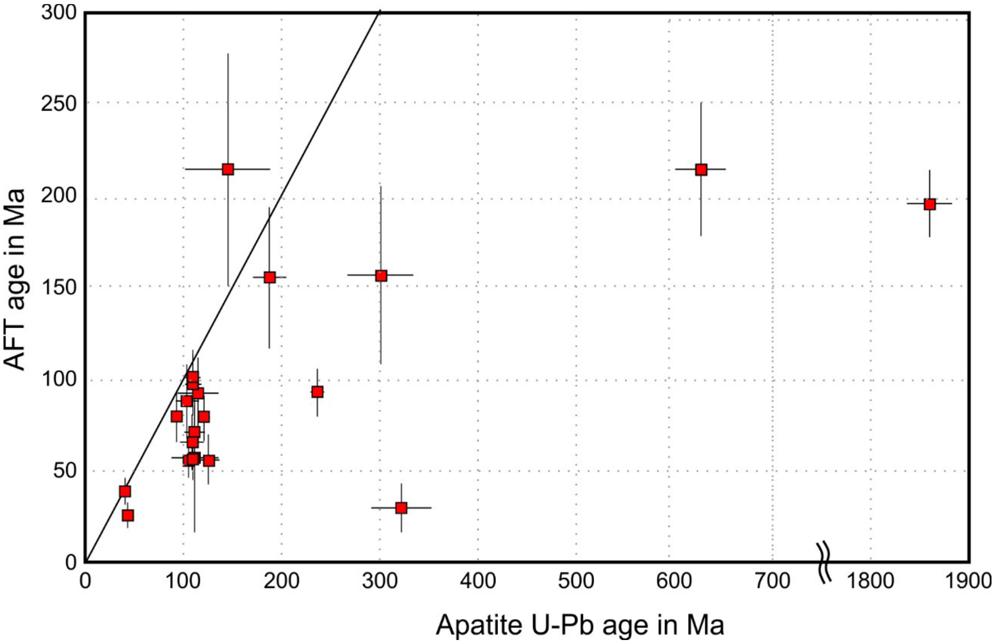

Fig. S9: Results of apatite U-Pb / FT double dating. The figure shows U-Pb ages vs AFT ages for all double-dated detrital apatite grains. Black line connects points of equal U-Pb and AFT age. U-Pb and AFT error bars are 2σ and 1σ, respectively.

The supplementary tables include:

- Table S1: The Science Team of Expedition PS104
- Table S2: Documentation and analytical information of individual samples analysed for this study.
- Table S3: Analytical details of LA-ICP-MS U-Pb, Lu-Hf isotopes and trace element measurements of detrital zircons contained in the sandstone of drill core PS104_20-2.
- Table S4: Analytical details of LA-ICP-MS U-Pb, Lu-Hf isotopes and trace element measurements of zircons contained in rhyolitic pebbles of drill core PS104_20-2.
- Table S5: Analytical details of LA-ICP-MS U-Pb and trace element measurements of zircons contained in rhyolitic bedrock from the Jones Mountains (Thurston Island Block).
- Table S6: Secondary standards measured during U-Pb analyses.
- Table S7: Analytical details of LA-ICP-MS U-Pb geochronology, Sm-Nd isotopes and trace elements measurements of detrital apatite contained in the sandstone of drill core PS104_20-2.
- Table S8: Analytical details of LA-ICP-MS U-Pb geochronology and trace elements measurements of detrital rutile contained in the sandstone of drill core PS104_20-2.
- Table S9: Standards measured during Hf and Nd isotopic analyses.
- Table S10: Analytical details of fission track analyses of detrital apatite contained in the sandstone of drill core PS104_20-2.

- Table S11: Results of XRD Analyses of rhyolitic bedrock from the Jones Mountains compared to rhyolitic pebbles contained in drill core PS104_20-2.
- Table S12: Compositional analysis of the clay fraction (<2 µm) contained in the drill core PS104_20-2.
- Table S13: Results of the biomarker analyses of the sandstone of drill core PS104_20-2.
- Table S14: Grain size distribution (sand, silt and clay) of the sedimentary rocks retrieved from drill core PS104_20-2.
- Table S15: Sphericity of grains <2 mm, contained in drill core PS104_20-2.
- Table S16: Convexity of grains <2 mm, contained in drill core PS104_20-2.
- Table S17: Aspect ratio of grains <2 mm, contained in drill core PS104_20-2

REFERENCES AND NOTES

1. J. Zachos, G. R. Dickens, R. E. Zeebe, An early Cenozoic perspective on greenhouse warming and carbon-cycle dynamics. *Nature* **451**, 279–283 (2008).

2. D. Wilson, S. Jamieson, P. Barrett, G. Leitchenkov, K. Gohl, R. Larter, Antarctic topography at the Eocene-Oligocene boundary. *Palaeogeogr. Palaeoclimatol. Palaeoecol.* **335-336**, 24–34 (2012).

3. K. Gohl, T. Freudenthal, C.-D. Hillenbrand, J. Klages, R. Larter, T. Bickert, S. Bohaty, W. Ehrmann, O. Esper, T. Frederichs, C. Gebhardt, K. Küssner, G. Kuhn, H. Pälicke, T. Ronge, P. Simoes Pereira, J. Smith, G. Uenzelmann-Neben, C. van de Flierdt, MeBo70 seabed drilling on a polar continental shelf: Operational report and lessons from drilling in the Amundsen Sea Embayment of West Antarctica. *Geochem. Geophys. Geosyst.* **18**, 4235–4250 (2017).

4. J. Klages, U. Salzmann, T. Bickert, C.-D. Hillenbrand, K. Gohl, G. Kuhn, S. Bohaty, J. Titschack, J. Müller, T. Frederichs, T. Bauersachs, W. Ehrmann, T. van de Flierdt, P. Simoes Pereira, R. Larter, G. Lohmann, I. Niezgodzki, G. Uenzelmann-Neben, M. Zundel, C. Spiegel, C. Mark. D. Chew. J. Francis, G. Nehrke, the Science Team of Expedition PS104, Temperate rainforests near the South Pole during peak Cretaceous warmth. *Nature* **580**, 81–86 (2020).

5. W. LeMasurier, C. Landis, Mantle-plume activity recorded by low-relief erosion surfaces in West Antarctica and New Zealand. *GSA Bull.* **108**, 1450–1466 (1996).

6. M. Zundel, C. Spiegel, F. Lisker, P. Monien, Post mid-Cretaceous tectonic and topographic evolution of western Marie Byrd Land, West Antarctica: Insights from apatite fission track and (U-Th-Sm)/He data. *Geochem. Geophys. Geosyst.* **20**, 5831–5848 (2019).

7. S. Rocchi, W. LeMasurier, G. Di Vincenzo, Oligocene to Holocene erosion and glacial history in Marie Byrd Land, West Antarctica, inferred from exhumation of the Dorrel Rock intrusive complex and from volcano morphologies. *GSA Bull.* **118**, 991–1005 (2006).

8. C. Spiegel, J. Lindow, P. Kamp, O. Meisel, S. Mukasa, F. Lisker, G. Kuhn, K. Gohl, Tectonomorphic evolution of Marie Byrd Land—Implications for Cenozoic rifting activity and

onset of West Antarctic glaciation. *Global Planet. Change* **145**, 98–115 (2016).

9. S. Cande, J. Stock, R. Müller, T. Ishihara, Cenozoic motion between east and west Antarctica. *Nature* **404**, 145–150 (2000).

10. T. Wilch, W. McIntosh, Eocene and Oligocene volcanism at Mount Petras, Marie Byrd Land: Implications for middle Cenozoic ice sheet reconstructions in West Antarctica. *Ant. Sci.* **12**, 477–491 (2000).

11. W. LeMasurier, Neogene extension and basin deepening in the West Antarctic rift inferred from comparisons with the East African rift and other analogs. *Geology* **36**, 247–250 (2008).

12. W. LeMasurier, D. Rex, Evolution of linear volcanic ranges in Marie Byrd Land, West Antarctica. *J. Geophys. Res.*, **94** B6, 7223–7236 (1989).

13. D. Elliot, "*The geological and tectonic evolution of the Transantarctic Mountains: A review*" in Antarctic Palaeoenvironments and Earth-Surface Processes, M. J. Hambrey, P. F. Barker, P. J. Barrett, V. Bowman, B. Davies, J. L. Smellie, M. Tranter, Eds. (Geological Society London, Special Publications 381, 2013).

14. J. Prenzel, F. Lisker, N. Monsees, M. Balestrieri, A. Läufer, C. Spiegel, Development and inversion of the Mesozoic Victoria Basin in the Terra Nova Bay (Transantarctic Mountains) derived from thermochronological data. *Gondw. Res.* **53**, 110–128 (2018).

15. R. Pankhurst, I. Millar, The Pre-Mesozoic magmatic history of the Thurston Island Crustal Block, West Antarctica. *J. Geophys. Res.* **98B7**, 11 835-11 849 (1993).

16. T. Bauersachs, J. Compaoré, E. Hopmans, L. Stal, S. Schouten, J. Sinninghe Damsté, Distribution of heterocyst glycolipids in cyanobacteria. *Phytochemistry* **70**, 2034–2039 (2009).

17. T. Bauersachs, J. Rochelmeier, L. Schwark, Seasonal lake surface water temperature trends reflected by heterocyst glycolipid-based molecular thermometers. *Biogeosciences* **12**, 3741–3751 (2015).

18. T. Bauersachs, J. Russell, T. Evans, A. Schwalb, L. Schwark, A heterocyte glycolipid-based calibration to reconstruct past continental climate change. *Nat. Commun.* **12**, 2406 (2021).

19. B. Naafs, D. McCormick, G. Inglis, R. Pancost, the T-Gres peat database collaborators, Archaeal and bacterial H-GDGTs are abundant in peat and their relative abundance is positively correlated with temperature. *Geochim. Cosmochim. Acta* **227**, 156–170 (2018).

20. G. Uenzelmann-Neben, K. Gohl, K. Hochmuth, U. Salzmann, R. Larter, C.-D. Hillenbrand, J. Klages, Deep water inflow slowed offshore expansion of the West Antarctic Ice Sheet at the Eocene-Oligocene transition. *Nat. Commun. Earth Environ.* **3**, 369 (2022).

21. K. Gohl, G. Uenzelmann-Neben, R. Larter, C.-D. Hillenbrand, K. Hochmuth, T. Kalberg, E. Weigelt, B. Davy, G. Kuhn, F. Nitsche, Seismic stratigraphic record of the Amundsen Sea Embayment shelf from pre-glacial to recent times: Evidence for a dynamic West Antarctic ice sheet. *Mar. Geol.* **344**, 115–131 (2013).

22. J. Lindow, P. Kamp, S. Mukasa, M. Kleber, F. Lisker, K. Gohl, G. Kuhn, C. Spiegel, Exhumation history along the eastern Amundsen Sea coast, West Antarctica, revealed by low-temperature thermochronology. *Tectonics* **35**, 2239–2257 (2016).

23. M. Zundel, C. Spiegel, A. Mehling, F. Lisker, C.-D. Hillenbrand, P. Monien, A. Klügel, Thurston Island (West Antarctica) between Gondwana subduction and continental separation: A multi-stage evolution revealed by apatite thermochronology. *Tectonics* **38**, 878–897 (2019).

24. D. Rysmans, P. Claeys, L. Derienmaeker, D. Maes, R. Finsy, M. Van Molle, Size and shape analysis of sedimentary grains by automated dynamic image analysis. *Particle Particle Syst. Charact.* **23**, 381–387 (2006).

25. K. Vos, N. Vanderberghe, K. Elsen, Surface textural analysis of quartz grains by scanning electron microscopy (SEM): From sample preparation to environmental interpretation. *Earth-Sci. Rev.* **128**, 93–104 (2014).

26. E. Hopmans, J. Weijers, E. Schefuß, L. Herfort, J. Sinninghe Damsté, S. Schouten, A novel proxy for terrestrial organic matter in sediments based on branched and isoprenoid tetraether

lipids. *Earth Planet. Sci. Lett.* **224**, 107–116 (2004).

27. Y. Wang, C. Lin, B. Zhang, H. Liu, Sedimentary evolution and controlling factors of Early-Mid Miocene Deltaic systems in the Northern Pearl River Mouth Basin, South China Sea. *Sci. Reports* **11**, 6134 (2021).

28. A. White, A. Blum, M. Schulz, D. Vivit, D. Stonestrom, M. Larsen, S. Murphy, D. Eberl, Chemical weathering in a tropical watershed, Luquillo Mountains, Puerto Rico: I. Long-term versus short-term weathering fluxes. *Geochim. Cosmochim. Acta* **62**, 209–226 (1998).

29. M. Baatsen, A. von der Heydt, M. Huber, M. Kliphuis, P. Bijl, A. Sluijs, H. Dijkstra, The middle to late Eocene greenhouse climate modelled using the CESM 1.0.5. *Clim. Past* **16**, 2573–2597 (2020).

30. S. Passchier, D. Ciarletta, T. Miriagos, P. Bijl, S. Bohaty, An Antarctic stratigraphic record of stepwise ice growth through the Eocene-Oligocene transition. *GSA Bull.* **129**, 318–330 (2017).

31. W. Huang, W. Keller, Dissolution of rock-forming silicate minerals in organic acids: Simulated first-stage weathering of fresh mineral surfaces. *Am. Mineral.* **55**, 2076–2094 (1970).

32. H. Mansurbeg, The use of diagenetic signatures to distinguish marine from continental deposits in Triassic-Jurassic sandstone reservoirs from the UK Central Graben. *Mar. Pet. Geol.* **79**, 188–200 (2017).

33. M. Van Wyk de Vries, R. Bingham, A. Hein, A new volcanic province: An inventory of subglacial volcanoes in West Antarctica. *Geol. Soc. London Spec. Publ.* **461**, SP461.7 (2018).

34. S. Rocchi, P. Armienti, M. D'Orazio, S. Tonarini, J. Wijbrans, G. Di Vincenzo, Cenozoic magmatism in the western Ross Embayment: Role of mantle plume versus plate dynamics in the development of the West Antarctic Rift System. *J. Geophy. Res.* **107**, 2195 (2002).

35. C. Yakymchuk, C. Siddoway, C. Fanning, R. McFadden, F. Korhonen, M. Brown, Anatectic reworking and differentiation of continental crust along the active margin of Gondwana: A zircon Hf–O perspective from West Antarctica. *Geol. Soc. London Spec. Publ.* **383**, 169–210 (2013).

36. C. Yakymchuk, C. Brown,M. Brown, C. Siddoway, C. Fanning, F. Korhonen, Paleozoic evolution of western Marie Byrd Land, Antarctica, *Geol. Soc. America Bull.* **127**, 1464–1484 (2015).

37. T. Riley, M. Flowerdew, R. Pankhurst, P. Leat, I. Millar, C. Fanning, M. Whitehouse, A revised geochronology of Thurston Island, West Antarctica, and correlations along the proto-Pacific margin of Gondwana. *Ant. Sci.* **29**, 47–60 (2017).

38. D. Nelson, J. Cottle, Long-term geochemical and geodynamic segmentation of the paleo-Pacific margin of Gondwana: Insight from the Antarctic and adjacent sectors. *Tectonics* **36**, 3229–3247 (2017).

39. G. Hagen-Peter, J. Cottle, A. Tulloch, S. Cox, Mixing between enriched lithospheric mantle and crustal components in a short-lived subduction-related magma system, Dry Valleys area, Antarctica: Insights from U-Pb geochronology, Hf isotopes, and whole-rock geochemistry. *Lithosphere* **7**, 174–188 (2015).

40. D. Elliot, C. Fanning, S. Mukasa, I. Millar, Hf- and O-isotope data from detrital and granitoid zircons reveal characteristics of the Permian–Triassic magmatic belt along the Antarctic sector of Gondwana. *Geosphere* **15**, 576–604 (2019).

41. R. Bomparola, C. Ghezzo, E. Belousova, W. Griffin, S. O'Reilly, Resetting of the U–Pb Zircon System in Cambro-Ordovician Intrusives of the Deep Freeze Range, Northern Victoria Land Antarctica, *J. Petrol.* **48**, 327–364 (2006).

42. D. Nelson, J. Cottle, The secular development of accretionary orogens: Linking the Gondwana magmatic arc record of West Antarctica, Australia and South America. *Gondwana Res.* **63**, 15–33 (2018).

43. M. Flowerdew, I. Millar, M. Curtis, A. Vaughan, M. Horstwood, M. Whitehouse, C. Fanning, Combined U-Pb geochronology and Hf isotope geochemistry of detrital zircons from early Paleozoic sedimentary rocks, Ellsworth-Whitmore Mountains block, Antarctica. *Geol. Soc. Am. Bull.* **119**, 275–288 (2007).

44. J. Goodge, I. Williams, P. Myrow, Provenance of Neoproterozoic and lower Paleozoic siliciclastic rocks of the central Ross orogen, Antarctica: Detrital record of rift-, passive-, and active-margin sedimentation. *GSA Bull.* **116**, 1253–1279 (2004).

45. G. Hagen-Peter, J. Cottle, Evaluating the relative roles of crustal growth versus reworking through continental arc magmatism: A case study from the Ross orogen, Antarctica. *Gondwana Res.* **55**, 153–166 (2018).

46. C. Adams, Geochronological studies of the Swanson Formation of Marie Byrd Land, West Antarctica, and correlation with Northern Victoria Land, East Antarctica, and South Island, New Zealand. *New Zeal. J. Geol. Geophys.* **29**, 345–358 (1986).

47. G. O'Sullivan, D. Chew, G. Kenny, I. Henrichs, D. Mulligan, The trace element composition of apatite and its application to detrital provenance studies. *Earth Sci. Rev.* **201**, 103044 (2020).

48. P. Vermeesch, A. Resentini, E. Garzanti, An R package for statistical provenance analysis. *Sediment. Geol.* **336**, 14–25 (2016).

49. J. Prenzel, F. Lisker, M. Elsner, R. Schöner, M. Balestrieri, A. Läufer, U. Berner, C. Spiegel, Burial and exhumation of the Eisenhower range, Transantarctic Mountains, based on thermochronological, sedimentary rock maturity and petrographic constraints. *Tectonophysics* **630**, 113–130 (2014).

50. J. Prenzel, F. Lisker, M. Balestrieri, A. Läufer, C. Spiegel, The Eisenhower Range, Transantarctic Mountains: Evaluation of qualitative interpretation concepts of thermochronological data. *Chem. Geol.* **352**, 176–187 (2013).

51. F. Lisker, A. Läufer, Thermochronological research in northern Victoria Land (Antarctica): A key to the pre-disintegration palaeogeography of Panthalassian Gondwana. *Polarforschung* **80**, 100–110 (2011).

52. V. Olivetti, F. Rossetti, M. Balestrieri, D. Pace, G. Cornamusini, F. Talarico, Variability in uplift, exhumation and crustal deformation along the Transantarctic Mountains front in southern Victoria Land, Antarctica. *Tectonophysics* **745**, 229–244 (2018).

53. M. Balestrieri, V. Olivetti, F. Rossetti, C. Gautheron, S. Cattò, M. Zattin, Topography, structural and exhumation history of the Admiralty Mountains region, northern Victoria Land, Antarctica. *Geosci. Front.* **11**, 1841–1858 (2020).

54. P. Fitzgerald, E. Stump, Cretaceous and Cenozoic episodic denudation of the Transantarctic Mountains, Antarctica: New constraints from apatite fission track thermochronology in the Scott Glacier region. *J. Geophys. Res. Solid Earth* **102**, 7747–7765 (1997).

55. P. Fitzgerald, A. Gleadow, Fission-track geochronology, tectonics and structure of the Transantarctic Mountains in northern Victoria Land Antarctica. *Chem. Geol.* **73**, 169–198 (1988).

56. P. Fitzgerald, The Transantarctic Mountains of southern Victoria Land: The application of apatite fission track analysis to a rift shoulder uplift. *Tectonics* **11**, 634–662 (1992).

57. P. Fitzgerald, Thermochronologic constraints on post-Paleozoic tectonic evolution of the central Transantarctic Mountains, Antarctica. *Tectonics* **13**, 818–836 (1994).

58. P. Fitzgerald, S. Baldwin, L. Webb, P. O'Sullivan, Interpretation of (U–Th)/He single grain ages from slowly cooled crustal terranes: A case study from the Transantarctic Mountains of southern Victoria Land. *Chem. Geol.* **225**, 91–120 (2006).

59. A. Gleadow, P. Fitzgerald, Uplift history and structure of the Transantarctic Mountains: New evidence from fission track dating of basement apatites in the Dry Valleys area, southern Victoria Land. *Earth Planet. Sci. Lett.* **82**, 1–14 (1987).

60. S. Miller, P. Fitzgerald, S. Baldwin, Cenozoic range-front faulting and development of the Transantarctic Mountains near Cape Surprise, Antarctica: Thermochronologic and geomorphologic constraints. *Tectonics* **29**, 2457 (2010).

61. M. Zattin, D. Pace, B. Andreucci, F. Rossetti, F. Talarico, Cenozoic erosion of the Transantarctic Mountains: A source-to-sink thermochronological study. *Tectonophysics* **630**, 158–165 (2014).

62. P. Fitzgerald, E. Stump, Early Cretaceous uplift in the Ellsworth Mountains of West

Antarctica. *Science* **254**, 92–94 (1991).

63. D. Craw, R. Findlay, Hydrothermal alteration of Lower Ordovician granitoids and Devonian Beacon Sandstone at Taylor Glacier, McMurdo Sound, Antarctica. *New Zeal. J. Geol. Geophys.* **27**, 465–475 (1984).

64. D. Sugden, S. Jamieson, The pre-glacial landscape of Antarctica. *Scottish Geogr. J.* **134**, 203–223 (2018).

65. V. Olivetti, M.-L. Balestrieri, D. Chew, L. Zurli, M. Zattin, D. Pace, F. Drakou, G. Cornamusini, M. Perotti, Ice volume variations and provenance trends in the Oligocene-early Miocene glaciomarine sediments of the Central Ross Sea, Antarctica (DSDP Site 270). *Global Planet. Change* **221**, 104042 (2023).

66. C. Baroni, V. Noti, S. Ciccacci, G. Righini, M. Salvatore, Fluvial origin of the valley system in northern Victoria Land (Antarctica) from quantitative geomorphic analysis. *GSA Bull.* **117**, 212–228 (2005).

67. M. Tankersley, H. Horgan, C. Siddoway, F. Caratori Tontini, K. Tinto, Basement topography and sediment thickness beneath Antarctica's Ross Ice Shelf. *Geophysics. Res. Letters* **49**, e2021GL097371 (2022).

68. M. Morlighem, E. Rignot, T. Binder, D. Blankenship, R. Drews, G. Eagles, O. Eisen, F. Ferraccioli, R. Forsberg, P. Fretwell, V. Goel, J. Greenbaum, H. Gudmundsson, J. Guo, V. Helm, C. Hofstede, I. Howat, A. Humbert, W. Jokat, N. Karlsson, W. Sang Lee, K. Matsuoka, R. Millan, J. Mouginot, J. Paden, F. Pattyn, J. Roberts, S. Rosier, A. Ruppel, H. Seroussi, E. Smith, D. Steinhage, B. Sun, M. van den Broeke, T. van Ommen, M. van Wessem, D. Young, Deep glacial troughs and stabilizing ridges unveiled beneath the margins of the Antarctic ice sheet. *Nat. Geosci.* **13**,132–137 (2020).

70. G. Paxman, S. Jamieson, K. Hochmuth, K. Gohl, M. Bentley, G. Leitchenkov, F. Ferraccioli, Reconstructions of Antarctic topography since the Eocene-Oligocene boundary. *Palaeogeogr. Palaeoclimatol. Palaeoecol.* **535**, 109346 (2019).

71. C. Scotese, N. Wright, PALEOMAP Paleodigital Elevation Models (PaleoDEMS) for the Phanerozoic (PALEOMAP Project, 2018); https://earthbyte.org/paleodem-resource-scotese-and-wright-2018/.

72. J. Coenen, R. Scherer, P. Baudoin, S. Warny, I. Castañeda, R. Askin, Paleogene marine and terrestrial development of the West Antarctic Rift System. *Geophys. Res. Lett.* **47**, e2019GL085281 (2020).

73. R. Pankhurst, S. Weaver, J. Bradshaw, B. Storey, T. Ireland, Geochronology and geochemistry of pre-Jurassic superterranes in Marie Byrd Land, Antarctica. *J. Geophysics. Res.* **103**, 2529–2547 (1998).

74. B. Dorschel, L. Hehemann, S. Viquerat, F. Warnke, S. Dreutter, Y. Tenberge, D. Accettella, L. Felipe Barrios, E. Bazhenova, J. Black, F. Bohoyo, C. Davey, L. De Santis, C. Escutia Dotti, A. Fremand, P. Fretwell, J. Gales, J. Gao, L. Gasperini, J. Greenbaum, J. Henderson Jencks, K. Hogan, J. Hong, M. Jakobsson, L. Jensen, J. Kool, S. Larin, R. Larter, G. Leitchenkov, B. Loubrieu, K. Mackay, L. Mayer, R. Millan, M. Morlighem, F. Navidad, F. Nitsche, Y. Nogi, C. Pertuisot, A. Post, H. Pritchard, A. Purser, M. Rebesco, E. Rignot, J. Roberts, M. Rovere, I. Ryzhov, C. Sauli, T. Schmitt, A. Silvano, J. Smith, H. Snaith, A. Tate, K. Tinto, P. Vandenbossche, P. Weatherall, P. Wintersteller, C. Yang, T. Zhang, J. Arndt, The international bathymetric chart of the Southern Ocean Version 2. *Sci. Data* **9**, 275 (2022).

75. Y. Shang, A. Kaakinen, C. Beets, M. Prins, Aeolian silt transport processes as fingerprinted by dynamic image analysis of the grain size and shape characteristics of Chinese loess and Red Clay deposits. *Sedim. Geol.* **375**, 36–48 (2018).

76. K. Futa, W. LeMasurier, Nd and Sr isotopic studies on Cenozoic mafic lavas from West Antarctica: Another source for continental alkali basalts. *Contrib. Mineral. Petrol.* **83**, 38–44 (1983).

77. S. Hart, J. Blusztajn, W. LeMasurier, D. Rex, Hobbs Coast Cenozoic volcanism: Implications for the West Antarctic Rift System. *Chem. Geol.* **139**, 223–248 (1997).

78. K. Panter, S. Hart, P. Kyle, J. Blusztanjn, T. Wilch, Geochemistry of Late Cenozoic basalts from the Crary Mountains: Characterization of mantle sources in Marie Byrd Land, Antarctica. *Chem. Geol.* **165**, 215–241 (2000).

79. S. Richard, C. Smith, D. Kimbrough, P. Fitzgerald, B. Luyendyk, M. McWilliams, Cooling history of the northern Ford Ranges, Marie Byrd Land, West Antarctica, *Tectonics* **13**, 837–857 (1994).

80. C. Adams, D. Seward, S. Weaver, Geochronology of Cretaceous granites and metasedimentary basement on Edward VII Peninsula, Marie Byrd Land, West Antarctica. *Ant. Sci.* **7**, 265–276 (1995).

81. D. Chew, J. Petrus, B. Kamber, U–Pb LA–ICPMS dating using accessory mineral standards with variable common Pb. *Chem. Geol.* **363**, 185–199 (2014).

82. C. Paton, J. Hellstrom, B. Paul, J. Woodhead, J. Hergt, Iolite: Freeware for the visualisation and processing of mass spectrometric data. *J. Anal. At. Spectrom* **26**, 2508–2518 (2011).

83. J. Petrus, B. Kamber, VizualAge: A novel approach to laser ablation ICP-MS U-Pb geochronology data reduction. *Geostand. Geoanal. Res.* **36**, 247–270 (2012).

84. K. Ludwig, User's manual for Isoplot 3.75: A geochronological Toolkit for Microsoft Excel. *Berkeley Geochronol. Cent. Spec. Publ.* **4**, 70 (2012).

85. L. Nasdala, F. Corfu, B. Schoene, S. Tapster, C. Wall, M. Schmitz, M. Ovtcharova, U. Schaltegger, A. Kennedy, A. Kronz, P. Reiners, Y.-H. Yang, F.-Y. Wu, S. Gain, W. Griffin, D. Szymanowski, C. Chanmuang, M. Ende, J. Valley, M. Spicuzza, B. Wanthanachaisaeng, G. Giester, GZ 7 and GZ 8—Two zircon reference materials for SIMS U-Pb geochronology. *Geostand. Geoanal. Res.* **42**, 431–457 (2018).

86. Sláma, J., J. Košler, D. Condon, J. Crowley, A. Gerdes, J. Hanchar, M. Horstwood, G. Morris, L. Nasdala, N. Norberg, U. Schaltegger, B. Schoene, M. Tubrett, M. Whitehouse, Plešovice zircon—A new natural reference material for U–Pb and Hf isotopic microanalysis. *Chem. Geol.* **249**, 1–35 (2008).

87. M. Wiedenbeck, P. Allé, F. Corfu, W. Griffin, M. Meier, F. Oberli, A. von Quadt, J. Roddick, W. Spiegel, Three natural zircon standards for U-Th-Pb, Lu-Hf, trace element and REE analyses. *Geostand. Geoanal. Res.* **19**, 1–23 (1995).

88. F. McDowell, W. McIntosh, K. Farley, A precise 40Ar–39Ar reference age for the Durango apatite (U–Th)/He and fission-track dating standard. *Chem. Geol.* **214**, 249–263 (2005).

89. B. Schoene, S. Bowring, U–Pb systematics of the McClure Mountain syenite: Thermochronological constraints on the age of the 40Ar/39Ar standard MMhb. *Contrib. Mineral. Petrol.* **151**, 615–630 (2006).

90. S. Thomson, G. Gehrels, J. Ruiz, R. Buchwaldt, Routine low-damage apatite U-Pb dating using laser ablation–multicollector–ICPMS. *Geochem. Geophys. Geosyst.* **13**, 3928 (2012).

91. J. Stacey, J. Kramers, Approximation of terrestrial lead isotope evolution by a two-stage model. *Earth Planet. Sci. Lett.* **26**, 207–221 (1975).

92. D. Chew, P. Sylvester, M. Tubrett, U-Pb and Th-Pb dating of apatite by LA-ICPMS. *Chem. Geol.* **280**, 200–216 (2011).

93. G. Luvizotto, T. Zack, H. Meyer, T. Ludwig, S. Triebold, A. Kronz, C. Münker, D. Stockli, S. Prowatke, S. Klemme, D. Jacob, H. von Eynatten, Rutile crystals as potential trace element and isotope mineral standards for microanalysis. *Chem. Geol.* **261**, 346–369 (2009).

94. G. Shi, X. Li, Q. Li, Z. Chen, J. Deng, Y. Liu, Z. Kang, E. Pang, Y. Xu, X. Jia, Ion microprobe U-Pb age and Zr-in-rutile thermometry of rutiles from the Daixian rutile deposit in the Hengshan Mountains, Shanxi Province, China. *Econ. Geol.* **107**, 525–535 (2012).

95. M. Mao, A. Rukhlov, S. Rowins, J. Spence, L. Coogan, Apatite trace element compositions: A robust new tool for mineral exploration. *Econ. Geol.* **111**, 1187–1222 (2016).

96. J. Woodhead, J. Hellstrom, J. Hergt, A. Greig, R. Maas, Isotopic and elemental imaging of geological materials by laser ablation inductively coupled plasma-mass spectrometry. *Geostand. Geoanal. Res.* **31**, 331–343 (2007).

97. G. Nowell, R. Parrish, Simultaneous acquisition of isotope compositions and parent/daughter ratios by non-isotope dilution-mode plasma ionisation multi-collector mass spectrometry (PIMMS). *Spec. Publ. Soc. Chem.* **267**, 298–310 (2001).

98. J. Woodhead, J. Hergt, A preliminary appraisal of seven natural zircon reference materials for in situ Hf isotope determination. *Geostand. Geoanal. Res.* **29**, 183–195 (2005).

99. U. Söderlund, P. Patchett, J. Vervoort, C. Isachsen, The 176Lu decay constant determined by Lu–Hf and U–Pb isotope systematics of Precambrian mafic intrusions. *Earth Planet. Sci. Lett.* **219**, 311–324 (2004).

100. A. Bouvier, J. Vervoort, P. Patchett, The Lu–Hf and Sm–Nd isotopic composition of CHUR: Constraints from unequilibrated chondrites and implications for the bulk composition of terrestrial planets. *Earth Planet. Sci. Lett.* **273**, 48–57 (2008).

101. Y.-H. Yang, F.-Y. Wu, J.-H. Yang, D. Chew, L.-W. Xie, Z.-Y. Chu, Y.-B. Zhang, C. Huang, Sr and Nd isotopic compositions of apatite reference materials used in U–Th–Pb geochronology. *Chem. Geol.* **385**, 35–55 (2014).

102. D. DePaolo, G. Wasserburg, Nd isotopic variations and petrogenetic models. *Geophys. Res. Lett.* **3**, 249–252 (1976).

103. S. Jacobsen, G. Wasserburg, Sm-Nd isotopic evolution of chondrites. *Earth Planet. Sci. Lett.* **50**, 139–155 (1980).

104. A. Gleadow, D. Belton, B. Kohn, R. Brown, Fission track dating of phosphate minerals and the thermochronology of apatite. *Rev. Mineral. Geochem.* **48**, 579–630 (2002).

105. D. Chew, R. Donelick, Combined apatite fission track and U-Pb dating by LA-ICP-MS and its application in apatite provenance analysis. *Mineral. Assoc. Canada Short Course* **42**, 219–247 (2012).

106. N. Cogné, D. Chew, R. Donelick, C. Ansberque, LA-ICP-MS apatite fission track dating: A practical zeta-based approach. *Chem. Geol.* **531**, 119302 (2019).

107. D. Chew, R. Donelick, M. Donelick, B. Kamber, M. Stock, Apatite chlorine concentration measurements by LA-ICP-MS. *Geostand. Geoanal. Res.* **38**, 23–35 (2014).

108. N. Roduit, *Two Complementary Efficient Methods to Quantify Porosity Types in Digital Images of Thin Sections With the Software JMicroVision*, Muscat, Oman, 10 to 13 January 2005 (24th IAS Meeting of Sedimentology, Scenic Sedimentology, 2005).

109. D. Moore, R. Reynolds, *X-ray Diffraction and the Identification and Analysis of Clay Minerals* (Oxford Univ. Press, 1989), 322 pp.

110. C. Vogt, J. Lauterjung, R. Fischer, Investigation of the clay fraction (<2 µm) of the clay minerals society reference clays. *Clays Clay Miner.* **50**, 388–400 (2002).

111. F. Chung. Quantitative interpretation of X-ray diffraction patterns of mixtures. I. Matrixflushing method for quantitative multicomponent analysis. J. Appl. Crystallogr. 7, 519–525 (1974).

112. W. Ehrmann, C.-D. Hillenbrand, J. Smith, A. Graham, G. Kuhn, R. Larter, Provenance

changes between recent and glacial-time sediments in the Amundsen Sea embayment, West Antarctica: Clay mineral assemblage evidence. Ant. Sci. 23, 471–486 (2011).

113. K. Weidenbach, L. Nickel, H. Neve, O. Alkhnbashi, S. Künzel, A. Kupczok, T. Bauersachs, L. Cassidy, A. Tholey, R. Backofen, R. Schmitz, Methanosarcina Spherical Virus, a novel archaeal lytic virus targeting Methanosarcina strains. J. Virol. 91, e00955–17 (2017).

114. D. Stalling, M. Westerhoff, H. Hege, "Amira: A highly interactive system for visual data analysis" in The Visualization Handbook C. D. Hansen, C. R. Johnson, Eds. (Elsevier, 2005), pp. 749–767.
